# Germline *BAP1* mutations induce a Warburg effect

**DOI:** 10.1038/cdd.2017.95

**Published:** 2017-06-30

**Authors:** Angela Bononi, Haining Yang, Carlotta Giorgi, Simone Patergnani, Laura Pellegrini, Mingming Su, Guoxiang Xie, Valentina Signorato, Sandra Pastorino, Paul Morris, Greg Sakamoto, Shafi Kuchay, Giovanni Gaudino, Harvey I Pass, Andrea Napolitano, Paolo Pinton, Wei Jia, Michele Carbone

**Affiliations:** 1 Thoracic Oncology Program, University of Hawaii Cancer Center, Honolulu, HI 96813, USA; 2 Department of Morphology-Surgery-Experimental Medicine, University of Ferrara, Ferrara, Italy; 3 Metabolomics Unit, University of Hawaii Cancer Center, Honolulu, HI 96813, USA; 4 Cancer Center, New York University, New York, NY 10016, USA

## Abstract

Carriers of heterozygous germline *BAP1* mutations (*BAP1*^+/−^) develop cancer. We studied plasma from 16 *BAP1*^+/−^ individuals from 2 families carrying different germline *BAP1* mutations and 30 *BAP1* wild-type (*BAP1*^*WT*^) controls from these same families. Plasma samples were analyzed by liquid chromatography time-of-flight mass spectrometry (LC-TOF-MS), ultra-performance liquid chromatography triple quadrupole mass spectrometry (UPLC-TQ-MS), and gas chromatography time-of-flight mass spectrometry (GC-TOF-MS). We found a clear separation in the metabolic profile between *BAP1*^*WT*^ and *BAP1*^+/−^ individuals. We confirmed the specificity of the data *in vitro* using 12 cell cultures of primary fibroblasts we derived from skin punch biopsies from 12/46 of these same individuals, 6 *BAP1*^+/−^ carriers and 6 controls from both families. *BAP1*^+/−^ fibroblasts displayed increased aerobic glycolysis and lactate secretion, and reduced mitochondrial respiration and ATP production compared with *BAP1*^*WT*^. siRNA-mediated downregulation of BAP1 in primary *BAP1*^*WT*^ fibroblasts and in primary human mesothelial cells, led to the same reduced mitochondrial respiration and increased aerobic glycolysis as we detected in primary fibroblasts from carriers of *BAP1*^+/−^ mutations. The plasma and cell culture results were highly reproducible and were specifically and only linked to *BAP1* status and not to gender, age or family, or cell type, and required an intact BAP1 catalytic activity. Accordingly, we were able to build a metabolomic model capable of predicting *BAP1* status with 100% accuracy using data from human plasma. Our data provide the first experimental evidence supporting the hypothesis that aerobic glycolysis, also known as the ‘Warburg effect’, does not necessarily occur as an adaptive process that is consequence of carcinogenesis, but rather that it may also predate malignancy by many years and facilitate carcinogenesis.

We discovered that inherited heterozygous germline mutations in the BRCA-associated protein 1 (*BAP1*) gene cause a high rate of mesothelioma and uveal melanoma.^1^ Our data, confirmed and expanded by others in several *BAP1*^+/−^ families across the world, revealed that germline *BAP1* mutations in addition to mesothelioma and uveal melanoma, also cause other malignancies.^2, 3, 4, 5^ We named this condition the ‘BAP1 cancer syndrome’.^2, 6^ So far, all individuals affected by this novel cancer syndrome developed at least one and often multiple cancers during their lifetime.^7^ Intriguingly, most *BAP1*^+/−^ patients experience significantly longer survival compared with patients who develop these same malignancies sporadically.^7, 8, 9^

BAP1 is a deubiquitylase that regulates multiple activities^10, 11, 12, 13, 14, 15, 16^ by forming multi-protein complexes.^6^ BAP1 stabilizes PGC-1*α* and promotes gluconeogenesis.^17^ Dey’s team found that the glucose and hexose metabolic pathways were repressed in liver-specific *Bap1* knockout mice.^18^ These findings^17, 18^ suggest that BAP1 may influence cellular metabolism. Very recently, we discovered a novel BAP1 cytoplasmic activity that, in concert with its nuclear activities, allows BAP1 to modulate gene–environment interaction and contributes to the high incidence of cancer in *BAP1*^+/−^ carriers.^19^

Adult ‘normal’ differentiated cells derive energy mostly via oxidative respiration (the tricarboxylic acid (TCA) cycle and oxidative phosphorylation). Alterations in the major cellular pathways involved in energy production – glycolysis and TCA cycle coupled with oxidative phosphorylation – are frequently found in cancer cells.^20^ Warburg’s findings^21^ that cancer cells obtain approximately the same amount of energy from fermentation (glycolysis to lactic acid) as from the TCA cycle is considered a hallmark of malignancy.^20, 22, 23, 24, 25^ This phenomenon referred as the ‘Warburg effect’ protects tumor cells from hypoxia and provides a rich source of precursors for the biosynthesis of nucleic acid, fatty acids, and phospholipids that sustain tumor cell growth. Moreover, the lactic acid secreted in the extracellular space promotes tumor cell growth and protects tumor cells from immune cells.^20, 22, 23, 24^ As to the emergence of the Warburg effect, the prevalent hypothesis is that the hypoxia that characterizes the initial growth of premalignant lesions provides micro-environmental selection forces that facilitate the growth of cell phenotypes that can adapt to this harsh environment through resistance to hypoxia and micro-environmental acidosis.^26^ Thus adaptation to hypoxia via a Warburg effect and subsequent selection of those cell clones capable of surviving acidosis leads over time to malignant cell populations with a powerful growth advantage.^22^ In addition to tumor cells, embryonic and adult stem cells may also produce energy via aerobic glycolysis.^20^ Similarly, within the tumor microenvironment, activated immune cells (macrophages, dendritic cells, and T cells) can also use aerobic glycolysis that helps them survive the hypoxic tumor environment.^27, 28^

Here we show that cells from *BAP1*^+/−^ carriers do not need to go through a selection process that favors the emergence of clones with a Warburg effect required for tumor growth: normal primary *BAP1*^+/*−*^ cells constitutionally derive a large part of their energy through aerobic glycolysis.

## Results

### Individuals carrying heterozygous germline *BAP1* mutations can be reliably identified based on their metabolic profile

We analyzed the metabolite profiles of plasma samples from 46 members of two unrelated families carrying different germline *BAP1* mutations – the Wisconsin (W) and the Louisiana (L) families – that we have been following and treating for mesothelioma and other malignancies for >10 years.^1^ For details about individuals and samples, see Materials/Subjects and Methods section^19^ and Supplementary Table S1.

**Figure 1:**
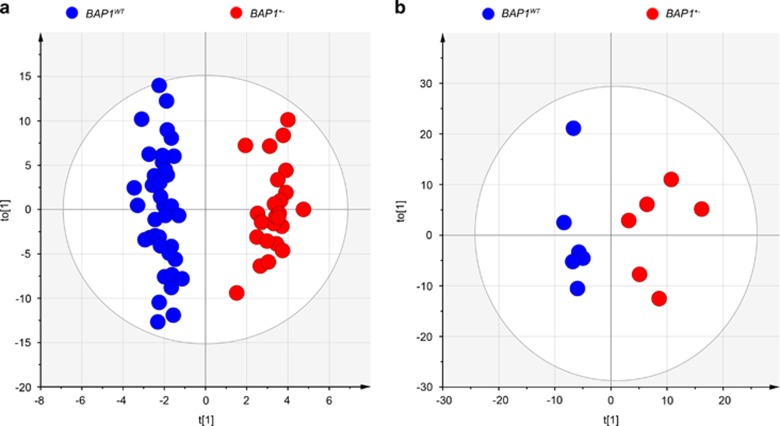
OPLS-DA analysis of metabolite profiles showed a global metabolic difference between *BAP1*^*WT*^ and *BAP1*^+/−^ individuals. (**a**) OPLS-DA score plot derived from, UPLC-TQ-MS and GC-TOF-MS spectral data of *N*=61 plasma samples (37 *BAP1*^*WT*^ (blue dots) and 24 *BAP1*^+/−^ (red dots)), *K*=412 variables, including identified metabolites (246) and unknowns. (**b**) OPLS-DA score plot of UPLC-TQ-MS and GC-TOF-MS spectral data of whole-cell extract samples from *BAP1*^*WT*^ (*N*=6) and *BAP1*^+/−^ (*N*=6) fibroblast cell cultures, *K*=495 variables, including identified metabolites (226) and unknowns

**Figure 2:**
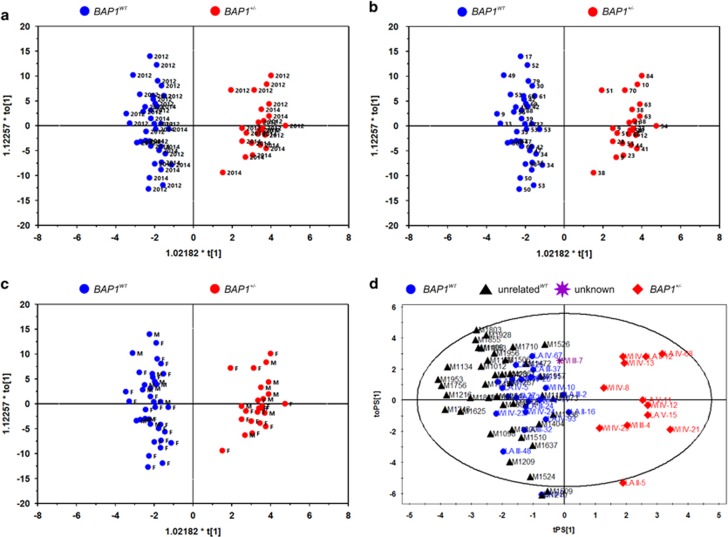
Genotype of *BAP1*^*WT*^ and *BAP1*^+/−^ individuals can be predicted from plasma samples using an OPLS-DA analysis of their metabolite profile independently of year of collection, age, gender, or family of origin. (**a**–**c**) OPLS-DA analysis of metabolite profiles is not influenced by (**a**) the year of collection (2012 and 2014, displayed in the panel), (**b**) the age of the individuals, and (**c**) gender (M=male, F=female). (**d**) OPLS-DA analysis differentiates *BAP1*^+/−^ carriers independently of family of origin. LC-TOF-MS and GC-TOF-MS were used for the metabolomic profiles of plasma samples from 22 *BAP1*^*WT*^ subjects (blue dots), 12 *BAP1*^+/−^ (red diamonds), 41 healthy controls unrelated to the W and L families (black triangles), and 1 individual of the W family with unknown *BAP1* status at the time the analysis was performed – later confirmed to be *BAP1*^*WT*^ – (purple star)

Plasma and fibroblast^19^ extracts were analyzed by liquid chromatography time-of-flight mass spectrometry (LC-TOF-MS), ultra-performance liquid chromatography triple quadrupole mass spectrometry (UPLC-TQ-MS), and gas chromatography time-of-flight mass spectrometry (GC-TOF-MS). A multivariate statistical analysis of the identified metabolites (Table 1), orthogonal partial least squares discriminant analysis (OPLS-DA), revealed a clear and statistically significant separation between *BAP1*^*WT*^ and *BAP1*^+/−^ individuals, both in plasma (OPLS-DA model: R2X=0.208, R2Y(cum)=0.951, Q2(cum)=0.313) (Figure 1a) and in cell extracts (OPLS-DA model: R2X=0.321, R2Y(cum)=0.916, Q2(cum)=0.215) (Figure 1b). The OPLS-DA model was not influenced by the year of collection of the samples (Figure 2a) and the age (Figure 2b) or gender (Figure 2c) of the individuals, suggesting that the differences observed in Figure 1a were specifically related to *BAP1* status (Figure 2 and Supplementary Figure S1). To test whether the OPLS-DA model established with the metabolomics data of human subjects with known *BAP1* status was able to clearly discriminate among samples from individuals with or without heterozygous germline *BAP1* mutations (Figure 2d), we analyzed blindly plasma samples from 76 individuals: 22 *BAP1*^*WT*^, 12 *BAP1*^+/−^, 1 individual of the W family with unknown *BAP1* status at the time the analysis was performed, and 41 healthy controls not related to the W and L families. The OPLS-DA model based on plasma metabolomics data was able to clearly discriminate the 41 healthy controls as *BAP1*^*WT*^ (Figure 2d). The *BAP1* unknown sample (WI III-7) was classified among the *BAP1*^*WT*^, a finding later confirmed by genomic testing (Figure 2d and Supplementary Figure S1).

Variable importance in the projection (VIP) values were calculated to evaluate the contribution of individual metabolite to the OPLS-DA model. Large VIP values >1.0 are the most relevant for explaining differences between *BAP1*^*WT*^ and *BAP1*^+/−^ groups. A total of 71 metabolites identified in plasma samples had VIP>1, among which 28 had VIP values >1.5 (Table 1 and Supplementary Table S2). A total of 111 metabolites identified in samples from cells in tissue culture had VIP>1, among which 21 had VIP values >1.5 (Table 1 and Supplementary Table S3). To determine how many metabolites were increased/decreased in the *BAP1*^+/−^ samples, we calculated the fold change as the ratio *BAP1*^+/−^/*BAP1*^*WT*^ of the average metabolite amount (Supplementary Table S2 and Supplementary Table S3).

### Germline *BAP1* mutations have significant effects on energy metabolism

**Figure 3:**
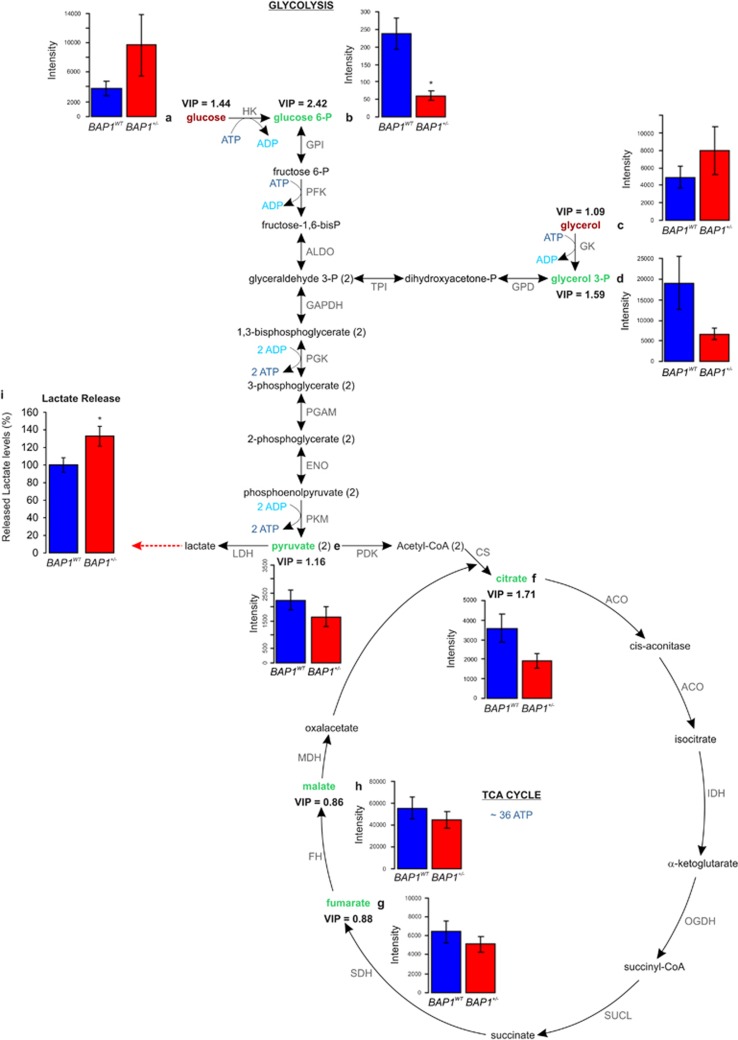
Altered metabolic pathways for the most relevant distinguishing metabolites between *BAP1*^*WT*^ and *BAP1*^+/−^ cells. Glycolysis converts glucose into pyruvate. In the presence of oxygen, in the mitochondria, pyruvate is completely oxidized and the energy is stored as ATP (aerobic metabolism). In the absence of sufficient oxygen or in tumor cells because of the Warburg effect, the pyruvate is reduced to lactate (anaerobic metabolism). (**a**–**h**) Levels of glucose (**a**), glucose 6-P (**b**), glycerol (**c**), glycerol 3-P (**d**), pyruvate (**e**), citrate (**f**), fumarate (**g**), and malate (**h**) were measured in whole-cell extract samples from *BAP1*^*WT*^ and *BAP1*^+/−^ fibroblast cell cultures. Bar plots show the mean±S.E.M. of the average metabolite intensity. Our analysis (see also Supplementary Table S3) revealed decreased levels of glucose 6-P (VIP=2.42), glycerol 3-P (VIP=1.59), pyruvate (VIP=1.16), and citrate (VIP=1.71) in *BAP1*^+/−^ fibroblasts. A slight reduction in the levels of other TCA cycle intermediates – fumarate and malate – was also found. Glucose (VIP=1.44) and glycerol (VIP=1.09) levels were increased in *BAP1*^+/−^ fibroblasts compared with *BAP1*^*WT*^. *P<0.05. (**i**) Basal lactate secretion is increased in *BAP1*^+/−^ fibroblasts compared with *BAP1*^*WT*^. The amount of lactate released (dotted red arrow) in the cell culture media was determined using a colorimetric assay; data shown as mean±S.E.M. of three independent experiments; *P<0.05. ACO, aconitase; ADP, Adenosine diphosphate; ALDO, aldolase; ATP, adenosine triphosphate; CS, citrate synthase; ENO, enolase; FH, fumarate hydratase; GPD, glycerol-3-phosphate dehydrogenase; GAPDH, glyceraldehyde-3-phosphate dehydrogenase; GPI, glucose-6-phosphate isomerase; IDH, isocitrate dehydrogenase; LDH, lactate dehydrogenase; MDH, malate dehydrogenase; PDK, pyruvate dehydrogenase kinase; PFK, phosphofructokinase; PGAM, phosphoglycerate mutase; PGK, phosphoglycerate kinase; PKM, pyruvate kinase; OGDH, oxoglutarate (alpha-ketoglutarate) dehydrogenase; SUCL, succinate-CoA ligase; SDH, succinate dehydrogenase; TPI, triosephosphate isomerase

Several of the altered metabolites between *BAP1*^+/−^ and *BAP1*^*WT*^ were glycolytic or TCA cycle intermediates (Figures 3a–h). Among the cell metabolites with a VIP ≥1.5 (Supplementary Table S3 and Supplementary Figure S2), we found that glucose 6-phosphate (Figure 3b) was significantly decreased in *BAP1*^+/−^ samples (VIP=2.42, *P*=0.00972). Glycerol 3-phosphate (Figure 3d) and citrate (Figure 3f) were also decreased in *BAP1*^+/−^ samples, although the group difference was not significant by Student’s *t*-test (Supplementary Table S3). By extending the analysis to include the metabolites with VIP ≥1 (Supplementary Table S3), we found decreased levels of pyruvate (Figure 3e), while glucose (Figure 3a) and glycerol (Figure 3c) levels were increased in *BAP1*^+/−^ samples. A small reduction was detected in fumarate (Figure 3g) and malate (Figure 3h) levels in *BAP1*^+/−^ samples. Significantly increased lactate concentrations were detected in the conditioned cell culture medium of *BAP1*^+/−^ fibroblast cell cultures (+32.75%±11.24) compared with *BAP1*^*WT*^ (Figure 3i). Together, these findings suggested increased glycolysis and reduced aerobic mitochondrial respiration. However, the metabolomics data (Figure 3) might also fit the hypothesis of decreased hexokinase (HK) conversion (because the substrate metabolite, namely, glucose accumulates and the product metabolite, namely, glucose 6-phosphate decreases) and decreased glycerol kinase (GK) activity (because the substrate metabolite, namely, glycerol accumulates and the product metabolite, namely, glycerol 3-P decreases).

### Metabolic rate analysis using ^13^C-glucose confirms increased glucose consumption and lactate secretion in *BAP1*^*+/−*^ cells

**Figure 4:**
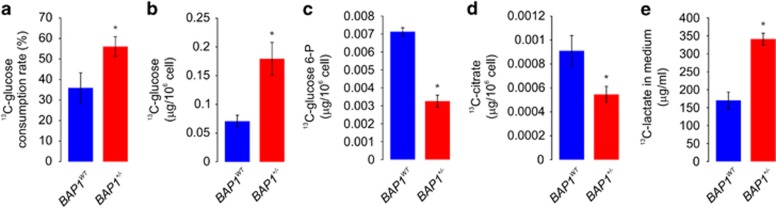
Levels of ^13^C-metabolites in culture medium and cells after culturing *BAP1*^*WT*^ and *BAP1*^+/−^ cells with ^13^C-glucose for 24 h. *BAP1*^*WT*^ and *BAP1*^+/−^ fibroblasts were incubated in ^13^C-glucose medium. (**a**) ^13^C-glucose consumption rate measured in tissue culture medium from *BAP1*^*WT*^ and *BAP1*^+/−^ cells. (**b**–**d**) ^13^C incorporation into ^13^C-glucose (**b**), ^13^C-glucose 6-P (**c**), and ^13^C-citrate (**d**) measured in cell extracts from *BAP1*^*WT*^ and *BAP1*^+/−^ cells. (**e**) Extracellular concentration of ^13^C-lactate produced by *BAP1*^*WT*^ and *BAP1*^+/−^ fibroblasts after 24 h of cell culture using ^13^C-glucose medium. Bar plots show the mean±S.E.M.; *P<0.05

Stable isotope (^13^C) tracking of central metabolic pathways provides a dynamic picture of metabolism and allows the experimental quantification of the integrated responses of metabolic networks.^29^ We performed comparative profiling of the ^13^C-labeled metabolic pattern in *BAP1*^*WT*^ and *BAP1*^+/−^ fibroblasts. After growing fibroblasts in ^13^C-labeled glucose, we performed a metabolic rate analysis based on UPLC-QTOFMS tracing pattern of the stable isotope in metabolites in a steady state (Figure 4). Analysis of ^13^C-glucose levels in conditioned medium revealed a significantly increased consumption rate in *BAP1*^+/−^ fibroblasts compared with *BAP1*^*WT*^ (Figure 4a). Analyses of cells extracts collected in parallel revealed increased levels of ^13^C-glucose in *BAP1*^+/−^ fibroblasts compared with *BAP1*^*WT*^ (Figure 4b). We also observed a reduction in intracellular ^13^C-glucose 6-P (Figure 4c) and ^13^C-citrate (Figure 4d) in *BAP1*^+/−^ fibroblasts, supporting the data shown in Figure 3 and Supplementary Table S3. Moreover, increased ^13^C-lactate concentrations were detected in the conditioned cell culture medium of *BAP1*^+/−^ fibroblasts compared with *BAP1*^*WT*^(Figure 4e). Thus, at the steady state, *BAP1*^+/−^ cells consumed more glucose (higher ^13^C-glucose consumption rate) and released more ^13^C-labeled lactate in the culture medium. At the same time, lower amounts of intracellular intermediates ^13^C-glucose 6-P and ^13^C-citrate were detected in *BAP1*^+/−^ cells. Mass distribution vectors^30^ of ^13^C-labeled glucose 6-P, citrate and lactate (Supplementary Table S4) showed that: glucose 6-P was only observed in form M+6; citrate was observed in seven forms: unlabeled and M+1, M+2, M+3, M+4, M+5, and M+6; lactate was observed in four forms: unlabeled, M+1, M+2, and M+3. Fractional contributions of ^13^C-labeled glucose 6-P and citrate were lower, while fractional contribution of lactate was higher in *BAP1*^+/−^ cells, confirming a faster glycolytic rate compared with *BAP1*^*WT*^ cells. These data indicate that *BAP1*^+/−^ cells have an increased glycolytic rate compared with *BAP1*^*WT*^ cells. *BAP1*^+/−^ cells take up more glucose because they rapidly metabolize it to lactate, which is then released extracellularly. A faster metabolic rate from glucose to extracellular lactate would quickly exhaust the glycolysis intermediates, explaining why *BAP1*^+/−^ cells have lower levels of ^13^C-glucose 6-P in spite of the increased glucose consumption rate. Thus the changes observed did not appear related to decreased HK or GK activity, an interpretation further supported by the experiments described below.

### *BAP1^+/−^* cells rely largely on the glycolytic metabolism for energy production

**Figure 5:**
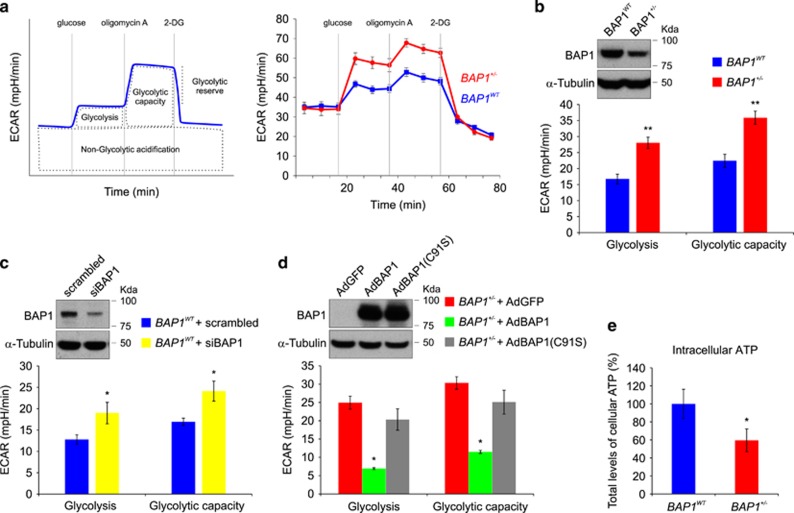
*BAP1*^+/−^ fibroblasts have increased glycolytic activity and decreased intracellular ATP. (**a**) ECAR was recorded over time in *BAP1*^*WT*^ and *BAP1*^+/−^ fibroblasts with the Seahorse XF96 extracellular flux analyzer. ECAR can be further dissected into different modules as depicted in the upper left scheme. The initial administration of a saturating concentration of glucose activates the glycolytic pathway producing NADH, protons, H_2_O, and ATP. Protons induce a rapid increase in ECAR. The following inhibition of mitochondrial ATP synthesis with Oligomycin A shifts the energy production to glycolysis and induces an additional increase in ECAR. Finally, inhibition of glycolysis with 2-deoxyglucose (2-DG) causes a strong decrease in ECAR levels, confirming that the ECAR observed was generated by the glycolytic activity of the cells. Representative experiments performed in *BAP1*^*WT*^ and *BAP1*^+/−^ fibroblasts are displayed on the right panel. (**b**) Levels of glycolysis and maximal glycolytic capacity in *BAP1*^*WT*^ and *BAP1*^+/−^ fibroblasts. The western blotting (WB) shows the amounts of WT BAP1 in total cell lysates of *BAP1*^*WT*^ and *BAP1*^+/−^ fibroblasts. (**c** and **d**) Rate of glycolysis and maximal glycolytic capacity in (**c**) *BAP1*^*WT*^ fibroblasts transfected with a pool of siRNAs targeting BAP1, and (**d**) *BAP1*^+/−^ fibroblasts transduced with adenoviruses for BAP1 (AdBAP1), its catalytic inactive mutant BAP1(C91S) (AdBAP1(C91S)), or GFP as control (AdGFP). In panel (**b**), the WB shows BAP1 protein levels in *BAP1*^*WT*^ fibroblasts silenced for BAP1. Fibroblasts were transfected with control scrambled siRNA or siBAP1 (a pool of four different siRNAs targeting BAP1: siBAP1#1, siBAP1#2, siBAP1#3, and siBAP1#5). In panel (**c**), the WB shows BAP1 protein levels in *BAP1*^+/−^ fibroblasts transduced with AdBAP1, catalytically inactive AdBAP1(C91S), or control (AdGFP). (**e**) Total levels of intracellular ATP are reduced in *BAP1*^+/−^ fibroblasts compared with *BAP1*^*WT*^. Bar plots show the mean±S.E.M. of three independent experiments; **P<0.01, *P<0.05

To further characterize the glycolytic pathway in *BAP1*^+/−^ cells, we measured the extracellular acidification rate (ECAR) in *BAP1*^*WT*^ and *BAP1*^+/−^ fibroblast cell cultures using the Seahorse XF96 Analyzer. When a saturating concentration of glucose (5 mM) is added, its catabolism to lactate through the glycolytic pathway produces ATP, protons, and thus a rapid increase in ECAR. We measured this glucose-induced response and reported it as the rate of glycolysis in basal conditions (Figures 5a and b). ECAR measurements (Figure 5b) revealed a significantly increased rate of glycolysis in *BAP1*^+/−^ fibroblast cell cultures (28.05±1.76 mpH/min) compared with *BAP1*^*WT*^ (16.73±1.52 mpH/min). The subsequent addition of the ATP synthase inhibitor – oligomycin A – inhibits mitochondrial ATP production and thus shifts the energy production to glycolysis. The consequent further increase in ECAR is a measure of the maximum glycolytic capacity of the cell. We found a significantly increased maximum glycolytic capacity in *BAP1*^+/−^ cells (35.88±2.05 mpH/min) compared with *BAP1*^*WT*^ (22.44±2.04 mpH/min) (Figure 5b).

Next, we silenced BAP1 in wild-type control fibroblasts using siRNAs (siBAP1) to test whether we were able to mimic the same increases in glycolysis and glycolytic capacity. ECAR measurements (Figure 5c) showed a significant increase in both parameters in BAP1-silenced cells (rate of glycolysis: *BAP1*^*WT*^ 12.76±1.12 mpH/min, siBAP1 18.98±2.51 mpH/min; glycolytic capacity: *BAP1*^*WT*^ 16.93±0.85 mpH/min, siBAP1 24.11±2.35 mpH/min). Similar results were obtained in primary human mesothelial (HM) cells, which we established in tissue culture from patients with benign pleural effusions treated with siBAP1 to reduce BAP1 expression levels (Supplementary Figures S3a and b).

We also transduced BAP1 in *BAP1*^+/−^ fibroblasts with adenoviruses for wild-type BAP1 (AdBAP1) or its catalytic inactive mutant carrying the C91S point mutation (AdC91S)^31^ or a control adenovirus (AdGFP). Figure 5d shows that *BAP1*^+/−^ fibroblasts transduced with AdBAP1 had reduced glycolysis and glycolytic capacity; in contrast, AdC91S was ineffective at reducing glycolysis (rate of glycolysis in *BAP1*^+/−^ transduced with: AdGFP 24.94±1.77 mpH/min, AdBAP1 6.92±0.24 mpH/min, AdBAP1(C91S) 20.33±2.92 mpH/min; glycolytic capacity: AdGFP 30.33±1.71 mpH/min, AdBAP1 11.50±0.43 mpH/min, AdC91S 25.08±3.25 mpH/min). These data further indicate that BAP1 regulates glycolysis and that *BAP1*^+/−^ cells rely largely on the aerobic glycolytic metabolism for energy production. Despite the enhanced glycolysis, we detected lower intracellular levels of total ATP in *BAP1*^+/−^ compared with *BAP1*^*WT*^ cells (Figure 5e). Conversion of glucose to lactate yields two ATP molecules; the subsequent TCA cycle generates ∼36 molecules of ATP.^32^ The reduced intracellular levels of ATP in *BAP1*^+/−^ cells suggested that mitochondrial respiration was impaired, and therefore *BAP1*^+/−^ cells were preferentially using glycolysis – rather than the TCA cycle – to produce ATP (Warburg effect).^21^

### *BAP1* mutations impair mitochondrial respiration

As citrate, a key TCA cycle intermediate, was decreased in carriers of germline *BAP1* mutations (Figures 3f and 4d), we sought to determine whether the boost in the glycolytic pathway of energy production observed in *BAP1*^+/−^ cells was associated with changes in mitochondrial respiration. First, we studied whether *BAP1*^+/−^ cells exhibited normal mitochondrial morphology and mitochondrial membrane potential (ΔΨ). Mitochondria were labeled in living cells by expressing green fluorescent protein specifically targeted to the mitochondria (mtGFP). mtGFP imaging (Supplementary Figure S4a) showed the normal three-dimensional interconnected tubular network characteristic of these organelles and no differences in mitochondrial number (Supplementary Figure S4b) or volume (Supplementary Figures S4c and d) between *BAP1*^*WT*^ and *BAP1*^+/−^ fibroblasts. Also no differences were detected in the loading of the fluorescent dye tetramethyl rhodamine methyl ester (TMRM) (Supplementary Figure S4e), thus ruling out ΔΨ differences between *BAP1*^*WT*^ and *BAP1*^+/−^ fibroblasts.

**Figure 6:**
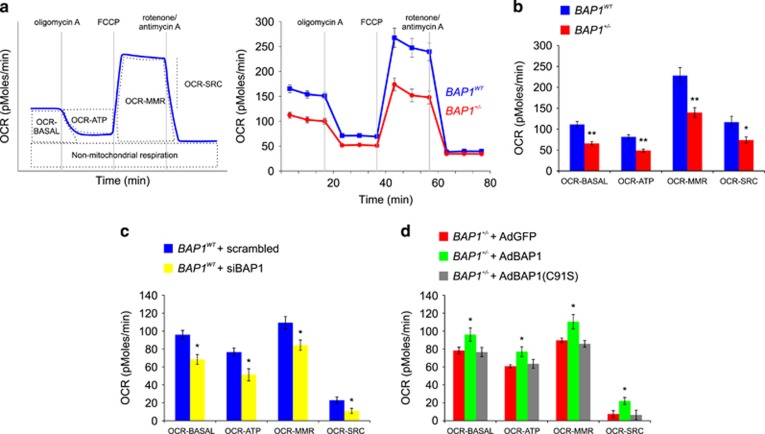
Mitochondrial respiratory function is impaired in *BAP1*^+/−^ cells. (**a**) OCR was analyzed in real time using the Seahorse XF96 extracellular flux analyzer. When Oligomycin A is added, the ATP synthase complex is inhibited, therefore the respiratory chain-associated oxygen consumption is inhibited. Addition of the ATP synthesis uncoupler FCCP induces the maximal oxygen consumption by the respiratory chain. Addition of rotenone and antimycinA (Rot/AntA, complex I and III inhibitors, respectively) blocks the electron transfer as well as oxygen consumption by the respiratory chain. Displayed on the left is a representative experiment showing the different parameters that can be measured and used to determine mitochondrial respiratory function: basal respiration (OCR-BASAL), ATP production (OCR-ATP), maximal respiration (OCR-MMR), and spare respiratory capacity (OCR-SRC). Representative experiments performed in *BAP1*^*WT*^ and *BAP1*^+/−^ fibroblasts are shown on the right. (**b**) Reduced OCR-BASAL, OCR-ATP, OCR-MMR, and OCR-SRC in *BAP1*^+/−^ fibroblasts compared with *BAP1*^*WT*^, indicating an overall impaired mitochondria-linked aerobic respiration and ATP production in germline mutated *BAP1*^+/−^ cells. (**c** and **d**) Levels of OCR-BASAL, OCR-ATP, OCR-MMR, and OCR-SRC in (**c**) *BAP1*^*WT*^ fibroblasts transfected with a pool of siRNAs targeting BAP1 and (**d**) *BAP1*^+/−^ fibroblasts transduced with adenoviruses (Ad) for BAP1 (AdBAP1), its catalytic inactive mutant AdBAP1(C91S), or GFP as control (AdGFP). Bar plots show the mean±S.E.M. of three independent experiments. **P<0.01, *P<0.05

Next, using the Seahorse XF96 Analyzer, we studied the oxygen consumption rate (OCR) in *BAP1*^*WT*^ and *BAP1*^+/−^ fibroblast cell cultures. The OCR is indicative of (and proportional to) mitochondrial respiration and can be employed as an indicator of mitochondrial respiratory capacity and energy production to reveal defects in mitochondrial bioenergetics mechanisms. The consecutive addition of specific mitochondrial inhibitors to the cells – Oligomycin A, FCCP, and rotenone/antimycin A – allows the measurements of distinct modules of oxygen consumption related to different mitochondrial processes (Figure 6a, left panel). Figure 6a (right panel) shows the OCR measurements over time in *BAP1*^*WT*^ and *BAP1*^+/−^ fibroblast cell cultures. *BAP1*^+/−^ fibroblasts exhibited significantly lower basal mitochondrial respiration (OCR-BASAL) (65.78±4.38 pMoles/min) compared with *BAP1*^*WT*^ (111.20±6.92 pMoles/min), supporting lower ATP turnover and demand (Figure 6b). The addition of Oligomycin A inhibits ATP synthase (complex V of the respiratory chain) causing a decrease in OCR that correlates with the amount of oxygen consumption linked to mitochondrial ATP production (OCR-ATP). ATP-linked respiration was significantly decreased in *BAP1*^+/−^ fibroblasts (48.86±3.38 pMoles/min) compared with *BAP1*^*WT*^ (81.48±5.24 pMoles/min) (Figure 6b). The maximal ATP output of mitochondria can be determined by addition of FCCP, an uncoupling agent that collapses the proton gradient and disrupts the mitochondrial membrane potential, inducing maximal oxygen consumption and substrate oxidation by complex IV (OCR-MMR, maximal mitochondrial respiration). OCR-MMR was significantly lower in *BAP1*^+/−^ fibroblasts (139.73±11.61 pMoles/min) compared with *BAP1*^*WT*^ (227.97±19.17 pMoles/min) (Figure 6b). The spare respiratory capacity (OCR-SRC) was determined by subtracting OCR-BASAL from FCCP-induced OCR-MMR. OCR-SRC measures the ability of the cells to respond to increased energy demand and was also significantly decreased in *BAP1*^+/−^ fibroblasts (73.94±7.51 pMoles/min) compared with *BAP1*^*WT*^ (116.77±14.00 pMoles/min) (Figure 6b). To summarize, in *BAP1*^+/−^ fibroblast cell cultures we observed an overall reduction in all the OCR parameters that quantify mitochondrial respiration.

To support these findings, we measured OCR parameters in *BAP1*^*WT*^ fibroblasts silenced for BAP1 (siBAP1). Similar to the *BAP1*^+/−^ cells (Figure 6b), we found that in siBAP1 cells all of the four critical parameters of mitochondrial respiration were significantly decreased: OCR-BASAL (*BAP1*^*WT*^ 95.89±4.90 pMoles/min, siBAP1 68.44±5.48 pMoles/min), OCR-ATP (*BAP1*^*WT*^ 76.63±4.55 pMoles/min, siBAP1 51.27±6.75 pMoles/min), OCR-MMR (*BAP1*^*WT*^ 109.27±6.91 pMoles/min, siBAP1 84.36±5.73 pMoles/min), and OCR-SCR (*BAP1*^*WT*^ 22.87±3.78 pMoles/min, siBAP1 10.91±2.91 pMoles/min) (Figure 6c). Similar results were obtained in primary HM cells in which BAP1 was silenced (Supplementary Figures S3a and c).

Next, we tested whether the rescue of BAP1 protein levels would be sufficient to revert the impairment in mitochondrial respiration. *BAP1*^+/−^ fibroblasts were transduced with AdGFP (control), AdBAP1, or AdBAP1(C91S). We found that the reintroduction of AdBAP1, but not of AdGFP or the catalytic inactive AdBAP1(C91S), was able to rescue mitochondrial respiration in *BAP1*^+/−^ cells, as determined by measurements of OCR-BASAL (AdGFP 78.29±3.82 pMoles/min, AdBAP1 96.10±7.42 pMoles/min, AdBAP1(C91S) 76.61±5.14 pMoles/min), OCR-ATP (AdGFP 60.81±1.56 pMoles/min, AdBAP1 77.07±5.40 pMoles/min, AdBAP1(C91S) 63.46±4.96 pMoles/min), OCR-MRR (AdGFP 89.70±2.57 pMoles/min, AdBAP1 110.43±7.95 pMoles/min, AdBAP1(C91S) 85.86±3.72 pMoles/min), OCR-SCR (AdGFP 7.46±3.81 pMoles/min, AdBAP1 22.02±3.90 pMoles/min, and AdBAP1(C91S) 6.53±5.26 pMoles/min) (Figure 6d).

Gene expression analysis of genes coding for enzymes participating in glycolysis, glycerol metabolism, the pentose phosphate pathway, glycogen metabolism, and TCA cycle revealed no significant transcriptional differences between *BAP1*^*WT*^ and *BAP1*^+/−^ fibroblasts (Supplementary Table S5). Together our findings indicate that the metabolomic changes detected are regulated posttranscriptionally and linked specifically to BAP1 deubiquitylase activity.

## Discussion

We discovered that normal primary cells carrying heterozygous germline *BAP1* mutations have increased aerobic glycolysis and impaired mitochondrial respiration. These metabolic changes in *BAP1*^+/−^ mutation carriers are so specific that, based on the metabolomics data from human plasma, we were able to predict *BAP1* status with 100% accuracy. The notion that genotype can be predicted by plasma metabolic analyses may seem implausible; however, the same results were reproducible regardless of the year of collection and gender or age of the individuals. Indeed, medium of fibroblast cell cultures derived from 12 of these same individuals retained many of the same metabolomics differences, underscoring the specificity of our findings.

We identified significant differences in glycolysis and TCA cycle metabolites between *BAP1*^+/−^ and *BAP1*^*WT*^ fibroblast cell cultures. *BAP1*^+/−^ cells consumed more glucose and released more lactate in the culture medium, indicative of a faster glycolytic rate compared with *BAP1*^*WT*^. These data indicate that *BAP1*^+/−^ cells rely largely on the glycolytic metabolism for energy production. Indeed, in comparison with *BAP1*^*WT*^, ECAR measurements in *BAP1*^+/−^ cells revealed a significantly increased rate of glycolysis, while we observed an overall reduction in all the OCR parameters that quantify mitochondrial respiration. ECAR was reduced and OCR restored in *BAP1*^+/−^ cells transduced with WT BAP1 (AdBAP1) but not with a catalytic inactive BAP1 (AdBAP1(C91S)). Moreover, similarly altered ECAR and OCR parameters were observed in *BAP1*^*WT*^ primary HM cells and fibroblasts silenced for BAP1, indicating that the effects observed are caused by the reduced BAP1 protein levels, independently of donor or cell type. Therefore, we anticipate that our results are also relevant to the numerous malignancies that carry somatic *BAP1* mutations.^33, 34, 35, 36, 37, 38, 39, 40, 41, 42, 43, 44^

The exact mechanism/s responsible for these metabolic changes is/are presently unknown. In a paper in press in *Nature*,^19^ we report that BAP1 modulates ER-to-mitochondria calcium (Ca^2+^) release.^19^ Intracellular Ca^2+^ modulates three rate-limiting enzymes of the TCA cycle.^45^ Ongoing studies in our laboratory will determine whether the deregulation of intracellular Ca^2+^ signaling is also responsible for the distinctive metabolic signature of *BAP1* mutation carriers.

The increased aerobic glycolysis – ‘Warburg effect’^20, 46^ – displayed by *BAP1*^+/−^ cells is frequently observed in cancer cells. In carriers of germline *BAP1* mutations, the presence of a Warburg effect in both the cells that undergo malignant transformation and in the surrounding stromal cells, creates an environment that promotes carcinogenesis and tumor growth.^20, 46^ Indeed, in all cell cultures from individuals carrying germline *BAP1* mutations, we observed increased levels of extracellular lactate. Lactate is known to promote cancer growth,^47^ stimulate angiogenesis,^48^ and polarize tumor-associated macrophages into a pro-tumor M2 phenotype.^49^ Alterations in cytokine levels and macrophage polarizations with a pronounced M2 phenotype were observed in the peritoneal cavity of asbestos-exposed *Bap1*^+/*−*^ mice and were linked to an increased incidence of MM.^50^ These findings support a model in which alterations in cellular metabolism due to reduced BAP1 levels may also induce immune system alterations associated with a microenvironment that favors malignant transformation.

In summary, our data indicate that the Warburg effect, in addition to being a hallmark of cancer cells,^25^ is also found in normal cells from individuals carrying heterozygous germline *BAP1* mutations and may contribute to the high incidence of cancer observed among them.

## Materials and methods

### Subjects

All participants (affected and unaffected family members) provided written informed consent according to the guidelines set forth by the Institutional Review Board of the University of Hawaii. Of the 14 individuals studied here from the W family, 7 were *BAP1*^*WT*^ and 7 were *BAP1*^+/−^ (all healthy at the time of sample collection; however, 2/7 *BAP1*^+/−^ individuals had been previously diagnosed with (a) MM and (b) with MM and Breast cancer, both in remission for >7 and >15 years, respectively). Of the 32 individuals from the L family, 23 were *BAP1*^*WT*^ and 9 were *BAP1*^+/−^ (all healthy at the time of sample collection, except 1/9 *BAP1*^+/−^ individuals diagnosed with uveal melanoma and MM 9 years prior to sample collection, who had stable disease at the time of blood collection). A total of 61 plasma samples were collected over a period of 2 years, 37 from *BAP1*^*WT*^ and 24 from *BAP1*^+/−^ individuals. We also studied 12 fibroblast cell cultures we established from skin punch biopsies from 6 *BAP1*^+/−^ carriers (3 from the W and 3 from the L family, all healthy at the time of sample collection, although 1 of them previously diagnosed with cancers 15 years prior, see above) and 6 *BAP1*^*WT*^ control individuals from the same families (3 from the W and 3 from the L family).^19^

### Cell cultures

Human dermal skin fibroblasts were derived from explants of skin biopsies of *BAP1*^*WT*^ and *BAP1*^+/−^ W and L families’ members.^19^ For details, see Supplementary Experimental Procedures.

### Plasma sample preparation and analysis by LC-TOF-MS

Metabolites were extracted from plasma samples and analyzed by LC-TOF-MS.

An Agilent HPLC 1200 system (Agilent Corporation, Santa Clara, CA, USA) was used with chromatographic separations performed on a 4.6 × 150 mm, 5 *μ*m Agilent ZORBAX Eclipse XDB-C18 chromatography column. Mass spectral data were acquired using an Agilent model 6220 MSD TOF mass spectrometer equipped with a dual sprayer electrospray ionization source (Agilent Corporation). Agilent API-TOF Reference Mass Solution Kit was used to obtain accurate mass time-of-flight data in both positive and negative mode operation. During metabolite profiling, both plot and centroid data were acquired for each sample from 50 to 1000 Da over a 25 min analysis time. Data generated by LC-TOF-MS were semiquantitative data and are expressed in peak intensity. For details, see Supplementary Experimental Procedures.

### Plasma sample preparation and analysis by UPLC-TQ-MS

Plasma samples were prepared as previously described with modifications.^51, 52, 53^ The supernatant was used for targeted metabolic profiling of 140 lipids and amino acids with an Acquity ultra performance liquid chromatography coupled to a Xevo TQ-S mass spectrometer (UPLC-TQ-MS, Waters Corp., Milford, MA, USA).^53^ Data generated by UPLC-TQ-MS were quantitative data and are expressed in μM.

### Plasma sample preparation and analysis by GC-TOF-MS

Metabolites extracted from plasma samples were analyzed using an Agilent 7890N gas chromatograph coupled with a Pegasus HT TOF mass spectrometer (Leco Corporation, St. Joseph, MI, USA). Electron impact ionization (70 eV) at full scan mode (*m*/*z* 40–600) was used, with an acquisition rate of 20 spectra/s in the TOF-MS setting. Data generated by GC-TOF-MS were semiquantitative data and are expressed in peak intensity. For details, see Supplementary Experimental Procedures.

### Cell extract preparation and analysis by UPLC-TQ-MS and GC-TOF-MS

*BAP1*^*WT*^ and *BAP1*^+/−^ fibroblasts were grown as described above, and 10^7^ cells were collected for the analysis.

Cell samples were prepared as previously described with modifications^52, 53^ as described in Supplementary Experimental Procedures. The supernatant was used for targeted metabolic profiling of 140 lipids with a UPLC-TQ-MS (Waters Corp., Milford, MA, USA)^53^ and, for untargeted metabolic profiling, with an Agilent 7890A gas chromatograph coupled to a Leco Pegasus time of flight mass spectrometer (GC-TOF-MS, Leco Corporation).^52^

### Data analysis

The acquired data files from LC-TOF-MS, UPLC-TQ-MS, and GC-TOF-MS were processed, combined, and analyzed using multivariate statistical tools to establish characteristic metabolic profiles associated with different genotypes. In total, 412 plasma and 495 cells’ chromatographic peaks corresponding to putative metabolites were found by deconvolution after exclusion of peaks originating from internal standards, contamination, and artifacts. Of these putative metabolites, 246 plasma metabolites and 226 cell metabolites were identified by their mass spectra and corresponding retention index (Table 1; see Supplementary Information).

### ^13^C-glucose metabolic pattern analysis

#### Cell culture, labeling, and sample collection

Fibroblasts were seeded at 5 × 10^5^ cells/T25 flask, in DMEM, 1 ×, 25 mM Glucose (Corning, Corning, NY, USA, Cat. No. 10-017-CV) supplemented with 10% Dialyzed FBS (Sigma, Cat. No. F0392). Labeling was started 24 h after seeding: cells were washed with phosphate-buffered saline (PBS) and culture medium was replaced with DMEM and no glucose (Gibco-Thermo Fisher Scientific, Waltham, MA, USA, Cat. No. 11966-025) supplemented with 10% Dialyzed FBS and 25 mM D-Glucose U-^13^C6 (Cambridge Isotope Laboratories, Inc., Tewksbury, MA, USA, Cat. No. 110187-42-3). A sample of the labeling media was taken at time zero and stored as a reference for analysis. ^13^C-labeled samples of medium and cells were collected following a 24-h labeling period. Cells were rinsed with PBS, detached with trypsin, and subjected to centrifugation at 200 × *g* for 5 min, at 4 °C. Cell pellets were washed three times with cold PBS, and the dry pellets were stored at −80 °C for subsequent analysis.

#### UPLC-QTOF-MS analysis

Cell culture medium samples were processed by adding 150 μl of acetonitrile to 50 μl of samples, while cell pellets (3–6 × 10^6^ cells) were extracted with 75% (v/v) methanol. After being vortexed for 10 min and centrifuged for 20 min at 16 100 × *g*, the supernatants (culture medium or cell extracts) were used for UPLC-QTOFMS analysis.

All analyses were performed on a Waters UPLC system (UPLC Acquity, Waters Corp., Manchester, UK) coupled with a quadrupole–time of flight mass spectrometer (Synapt G2, Waters Corp., Manchester, UK). Metabolites separation was achieved through a 2.1 × 100 mm 1.7 μm Acquity amide and an Acquity HSS C18 column (100 × 2.1 mm i.d., 1.7 μm; Waters Corp., Manchester, UK) equipped with ACQUITY UPLC VanGuard Pre-Column, separately, according to the published methods with modifications.^54^ The column was maintained at 40 °C and a 5 μl aliquot of sample was injected. The flow rate remained constant at 0.4 ml/min. UPLC-MS raw data obtained were analyzed using TargetLynx applications manager version 4.1 (Waters Corp., Manchester, UK). Quantification was achieved for each metabolite using linear regression analysis of the peak area of metabolite *versus* concentration.

### Gene silencing with siRNA, adenovirus-mediated gene transfer and transfection

siRNA oligonucleotides targeting four different BAP1 mRNAs and one negative control siRNA were obtained from Qiagen (Germantown, MD, USA) (GeneSoluton siRNA: Hs_BAP1_1, Cat. No.: SI00066696; Hs_BAP1_2, Cat. No.: SI00066703; Hs_BAP1_3, Cat. No.: SI00066710; Hs_BAP1_5, Cat. No.: SI03036390; AllStars Negative control siRNA, Cat. No.: 1027280). Tranfection was performed with HiPerfect (Qiagen), using a 10 nM final concentration of siRNA in 10 % FBS medium for 24 h.

The adenoviruses expressing BAP1 and GFP were purchased from SignaGen Laboratories (Rockville, MD, USA) (Ad-BAP1, Cat. No. SL175127; Ad-GFP, Cat. No. SL100708). A customized adenovirus expressing Ad-Myc-BAP1(C91S) was produced by SignaGen Laboratories using their Ad.MAX System.

### XF96 instrument setup and analysis

*BAP1*^*WT*^ and *BAP1*^+/−^ fibroblasts were seeded in XF96 Cell Culture Microplates. OCR and ECAR were measured simultaneously using the XF96 Analyzer (Seahorse Biosciences, North Billerica, MA, USA), and values were normalized to the number of cells per well using the crystal violet method. For details, see Supplementary Experimental Procedures.

## Figures and Tables

**Table 1:** Differentially expressed metabolites between *BAP1* ^*WT*^ and *BAP1* ^*+/−*^

	**Metabolomics profiling results**	
	**Plasma**	**Fibroblast cell cultures**
No. of total metabolites	412	495
No. of known metabolites	246	226
No. of metabolites with VIP≥1	71	111
No. of metabolites with VIP≥1.5	28	21

We found 412 plasma and 495 cells’ chromatographic peaks corresponding to putative metabolites; of these, 246 plasma metabolites and 226 cell metabolites could be identified. Metabolites with VIP≥1 are listed in Supplementary Tables S2 and S3

## References

[bib1] Testa JR, Cheung M, Pei J, Below JE, Tan Y, Sementino E et al. Germline BAP1 mutations predispose to malignant mesothelioma. Nat Genet 2011; 43: 1022–1025. 21874000 10.1038/ng.912 PMC3184199

[bib2] Carbone M, Ferris LK, Baumann F, Napolitano A, Lum CA, Flores EG et al. BAP1 cancer syndrome: malignant mesothelioma, uveal and cutaneous melanoma, and MBAITs. J Transl Med 2012; 10: 179. 22935333 10.1186/1479-5876-10-179 PMC3493315

[bib3] Wiesner T, Obenauf AC, Murali R, Fried I, Griewank KG, Ulz P et al. Germline mutations in BAP1 predispose to melanocytic tumors. Nat Genet 2011; 43: 1018–1021. 21874003 10.1038/ng.910 PMC3328403

[bib4] Popova T, Hebert L, Jacquemin V, Gad S, Caux-Moncoutier V, Dubois-d'Enghien C et al. Germline BAP1 mutations predispose to renal cell carcinomas. Am J Hum Genet 2013; 92: 974–980. 23684012 10.1016/j.ajhg.2013.04.012 PMC3675229

[bib5] Farley MN, Schmidt LS, Mester JL, Pena-Llopis S, Pavia-Jimenez A, Christie A et al. A novel germline mutation in BAP1 predisposes to familial clear-cell renal cell carcinoma. Mol Cancer Res 2013; 11: 1061–1071. 23709298 10.1158/1541-7786.MCR-13-0111 PMC4211292

[bib6] Carbone M, Yang H, Pass HI, Krausz T, Testa JR, Gaudino G. BAP1 and cancer. Nat Rev Cancer 2013; 13: 153–159. 23550303 10.1038/nrc3459 PMC3792854

[bib7] Baumann F, Flores E, Napolitano A, Kanodia S, Taioli E, Pass H et al. Mesothelioma patients with germline BAP1 mutations have 7-fold improved long-term survival. Carcinogenesis 2015; 36: 76–81. 25380601 10.1093/carcin/bgu227 PMC4291047

[bib8] Carbone M, Flores EG, Emi M, Johnson TA, Tsunoda T, Behner D et al. Combined genetic and genealogic studies uncover a large BAP1 cancer syndrome kindred tracing back nine generations to a common ancestor from the 1700s. PLoS Genet 2015; 11: e1005633. 26683624 10.1371/journal.pgen.1005633 PMC4686043

[bib9] Klebe S, Driml J, Nasu M, Pastorino S, Zangiabadi A, Henderson D et al. BAP1 hereditary cancer predisposition syndrome: a case report and review of literature. Biomark Res 2015; 3: 14. 26140217 10.1186/s40364-015-0040-5 PMC4488956

[bib10] Scheuermann JC, de Ayala Alonso AG, Oktaba K, Ly-Hartig N, McGinty RK, Fraterman S et al. Histone H2A deubiquitinase activity of the Polycomb repressive complex PR-DUB. Nature 2010; 465: 243–247. 20436459 10.1038/nature08966 PMC3182123

[bib11] Yu H, Mashtalir N, Daou S, Hammond-Martel I, Ross J, Sui G et al. The ubiquitin carboxyl hydrolase BAP1 forms a ternary complex with YY1 and HCF-1 and is a critical regulator of gene expression. Mol Cell Biol 2010; 30: 5071–5085. 20805357 10.1128/MCB.00396-10 PMC2953049

[bib12] Lee HS, Lee SA, Hur SK, Seo JW, Kwon J. Stabilization and targeting of INO80 to replication forks by BAP1 during normal DNA synthesis. Nat Commun 2014; 5: 5128. 25283999 10.1038/ncomms6128

[bib13] Yu H, Pak H, Hammond-Martel I, Ghram M, Rodrigue A, Daou S et al. Tumor suppressor and deubiquitinase BAP1 promotes DNA double-strand break repair. Proc Natl Acad Sci USA 2014; 111: 285–290. 24347639 10.1073/pnas.1309085110 PMC3890818

[bib14] Ismail IH, Davidson R, Gagne JP, Xu ZZ, Poirier GG, Hendzel MJ. Germline mutations in BAP1 impair its function in DNA double-strand break repair. Cancer Res 2014; 74: 4282–4294. 24894717 10.1158/0008-5472.CAN-13-3109

[bib15] Zarrizi R, Menard JA, Belting M, Massoumi R. Deubiquitination of gamma-tubulin by BAP1 prevents chromosome instability in breast cancer cells. Cancer Res 2014; 74: 6499–6508. 25228651 10.1158/0008-5472.CAN-14-0221

[bib16] Peng J, Ma J, Li W, Mo R, Zhang P, Gao K et al. Stabilization of MCRS1 by BAP1 prevents chromosome instability in renal cell carcinoma. Cancer Lett 2015; 369: 167–174. 26300492 10.1016/j.canlet.2015.08.013

[bib17] Ruan HB, Han X, Li MD, Singh JP, Qian K, Azarhoush S et al. O-GlcNAc transferase/host cell factor C1 complex regulates gluconeogenesis by modulating PGC-1alpha stability. Cell Metab 2012; 16: 226–237. 22883232 10.1016/j.cmet.2012.07.006 PMC3480732

[bib18] Baughman JM, Rose CM, Kolumam G, Webster JD, Wilkerson EM, Merrill AE et al. NeuCode proteomics reveals Bap1 regulation of metabolism. Cell Rep 2016; 16: 583–595. 27373151 10.1016/j.celrep.2016.05.096 PMC5546211

[bib19] Bononi A, Giorgi C, Patergnani S, Larson D, Verbruggen K, Tanji M et al. BAP1 regulates IP3R3-mediated Ca2+ flux to mitochondria suppressing cell transformation. Nature (in press; doi:10.1038/nature22798). 10.1038/nature22798 PMC5581194 28614305

[bib20] Cairns RA, Harris IS, Mak TW. Regulation of cancer cell metabolism. Nat Rev Cancer 2011; 11: 85–95. 21258394 10.1038/nrc2981

[bib21] Warburg O. On the origin of cancer cells. Science 1956; 123: 309–314. 13298683 10.1126/science.123.3191.309

[bib22] Gatenby RA, Gillies RJ. Why do cancers have high aerobic glycolysis? Nat Rev Cancer 2004; 4: 891–899. 15516961 10.1038/nrc1478

[bib23] Hsu PP, Sabatini DM. Cancer cell metabolism: Warburg and beyond. Cell 2008; 134: 703–707. 18775299 10.1016/j.cell.2008.08.021

[bib24] Koppenol WH, Bounds PL, Dang CV. Otto Warburg's contributions to current concepts of cancer metabolism. Nat Rev Cancer 2011; 11: 325–337. 21508971 10.1038/nrc3038

[bib25] Hanahan D, Weinberg RA. Hallmarks of cancer: the next generation. Cell 2011; 144: 646–674. 21376230 10.1016/j.cell.2011.02.013

[bib26] Suchorolski MT, Paulson TG, Sanchez CA, Hockenbery D, Reid BJ. Warburg and Crabtree effects in premalignant Barrett's esophagus cell lines with active mitochondria. PLoS ONE 2013; 8: e56884. 23460817 10.1371/journal.pone.0056884 PMC3584058

[bib27] O'Neill LA, Hardie DG. Metabolism of inflammation limited by AMPK and pseudo-starvation. Nature 2013; 493: 346–355. 23325217 10.1038/nature11862

[bib28] Biswas SK, Mantovani A. Orchestration of metabolism by macrophages. Cell Metab 2012; 15: 432–437. 22482726 10.1016/j.cmet.2011.11.013

[bib29] Zamboni N, Fendt SM, Ruhl M, Sauer U. (13)C-based metabolic flux analysis. Nat Protoc 2009; 4: 878–892. 19478804 10.1038/nprot.2009.58

[bib30] Buescher JM, Antoniewicz MR, Boros LG, Burgess SC, Brunengraber H, Clish CB et al. A roadmap for interpreting (13)C metabolite labeling patterns from cells. Curr Opin Biotechnol 2015; 34: 189–201. 25731751 10.1016/j.copbio.2015.02.003 PMC4552607

[bib31] Jensen DE, Proctor M, Marquis ST, Gardner HP, Ha SI, Chodosh LA et al. BAP1: a novel ubiquitin hydrolase which binds to the BRCA1 RING finger and enhances BRCA1-mediated cell growth suppression. Oncogene 1998; 16: 1097–1112. 9528852 10.1038/sj.onc.1201861

[bib32] Hinkle PC, Kumar MA, Resetar A, Harris DL. Mechanistic stoichiometry of mitochondrial oxidative phosphorylation. Biochemistry 1991; 30: 3576–3582. 2012815 10.1021/bi00228a031

[bib33] Pena-Llopis S, Vega-Rubin-de-Celis S, Liao A, Leng N, Pavia-Jimenez A, Wang S et al. BAP1 loss defines a new class of renal cell carcinoma. Nat Genet 2012; 44: 751–759. 22683710 10.1038/ng.2323 PMC3788680

[bib34] Harbour JW, Onken MD, Roberson ED, Duan S, Cao L, Worley LA et al. Frequent mutation of BAP1 in metastasizing uveal melanomas. Science 2010; 330: 1410–1413. 21051595 10.1126/science.1194472 PMC3087380

[bib35] Mori T, Sumii M, Fujishima F, Ueno K, Emi M, Nagasaki M et al. Somatic alteration and depleted nuclear expression of BAP1 in human esophageal squamous cell carcinoma. Cancer Sci 2015; 106: 1118–1129. 26081045 10.1111/cas.12722 PMC4582980

[bib36] Jiao Y, Pawlik TM, Anders RA, Selaru FM, Streppel MM, Lucas DJ et al. Exome sequencing identifies frequent inactivating mutations in BAP1, ARID1A and PBRM1 in intrahepatic cholangiocarcinomas. Nat Genet 2013; 45: 1470–1473. 24185509 10.1038/ng.2813 PMC4013720

[bib37] Nasu M, Emi M, Pastorino S, Tanji M, Powers A, Luk H et al. High incidence of somatic BAP1 alterations in sporadic malignant mesothelioma. J Thorac Oncol 2015; 10: 565–576. 25658628 10.1097/JTO.0000000000000471 PMC4408084

[bib38] Guo G, Chmielecki J, Goparaju C, Heguy A, Dolgalev I, Carbone M et al. Whole-exome sequencing reveals frequent genetic alterations in BAP1, NF2, CDKN2A, and CUL1 in malignant pleural mesothelioma. Cancer Res 2015; 75: 264–269. 25488749 10.1158/0008-5472.CAN-14-1008

[bib39] Lo Iacono M, Monica V, Righi L, Grosso F, Libener R, Vatrano S et al. Targeted next-generation sequencing of cancer genes in advanced stage malignant pleural mesothelioma: a retrospective study. J Thorac Oncol 2015; 10: 492–499. 25514803 10.1097/JTO.0000000000000436

[bib40] Bueno R, Stawiski EW, Goldstein LD, Durinck S, De Rienzo A, Modrusan Z et al. Comprehensive genomic analysis of malignant pleural mesothelioma identifies recurrent mutations, gene fusions and splicing alterations. Nat Genet 2016; 48: 407–416. 26928227 10.1038/ng.3520

[bib41] Joseph RW, Kapur P, Serie DJ, Parasramka M, Ho TH, Cheville JC et al. Clear cell renal cell carcinoma subtypes identified by BAP1 and PBRM1 expression. J Urol 2016; 195: 180–187. 26300218 10.1016/j.juro.2015.07.113 PMC5221690

[bib42] Chan-On W, Nairismagi ML, Ong CK, Lim WK, Dima S, Pairojkul C et al. Exome sequencing identifies distinct mutational patterns in liver fluke-related and non-infection-related bile duct cancers. Nat Genet 2013; 45: 1474–1478. 24185513 10.1038/ng.2806

[bib43] Wang Y, Thomas A, Lau C, Rajan A, Zhu Y, Killian JK et al. Mutations of epigenetic regulatory genes are common in thymic carcinomas. Sci Rep 2014; 4: 7336. 25482724 10.1038/srep07336 PMC4258655

[bib44] Yoshikawa Y, Emi M, Hashimoto-Tamaoki T, Ohmuraya M, Sato A, Tsujimura T et al. High-density array-CGH with targeted NGS unmask multiple noncontiguous minute deletions on chromosome 3p21 in mesothelioma. Proc Natl Acad Sci USA 2016; 113: 13432–13437. 27834213 10.1073/pnas.1612074113 PMC5127333

[bib45] Denton RM. Regulation of mitochondrial dehydrogenases by calcium ions. Biochim Biophys Acta 2009; 1787: 1309–1316. 19413950 10.1016/j.bbabio.2009.01.005

[bib46] DeBerardinis RJ, Chandel NS. Fundamentals of cancer metabolism. Sci Adv 2016; 2: e1600200. 27386546 10.1126/sciadv.1600200 PMC4928883

[bib47] Romero-Garcia S, Moreno-Altamirano MM, Prado-Garcia H, Sanchez-Garcia FJ. Lactate contribution to the tumor microenvironment: mechanisms, effects on immune cells and therapeutic relevance. Front Immunol 2016; 7: 52. 26909082 10.3389/fimmu.2016.00052 PMC4754406

[bib48] Vegran F, Boidot R, Michiels C, Sonveaux P, Feron O. Lactate influx through the endothelial cell monocarboxylate transporter MCT1 supports an NF-kappaB/IL-8 pathway that drives tumor angiogenesis. Cancer Res 2011; 71: 2550–2560. 21300765 10.1158/0008-5472.CAN-10-2828

[bib49] Colegio OR, Chu NQ, Szabo AL, Chu T, Rhebergen AM, Jairam V et al. Functional polarization of tumour-associated macrophages by tumour-derived lactic acid. Nature 2014; 513: 559–563. 25043024 10.1038/nature13490 PMC4301845

[bib50] Napolitano A, Pellegrini L, Dey A, Larson D, Tanji M, Flores EG et al. Minimal asbestos exposure in germline BAP1 heterozygous mice is associated with deregulated inflammatory response and increased risk of mesothelioma. Oncogene 2016; 35: 1996–2002. 26119930 10.1038/onc.2015.243 PMC5018040

[bib51] Bononi I, Perri P, Begnardi A, Martini A, Mazzoni E, Bosi S et al. Antibodies reacting with Simian Virus 40 capsid protein mimotopes in serum samples from patients affected by uveal melanoma. J Hematol Oncol 2014; 7: 38. 24886631 10.1186/1756-8722-7-38 PMC4012707

[bib52] Qiu Y, Cai G, Zhou B, Li D, Zhao A, Xie G et al. A distinct metabolic signature of human colorectal cancer with prognostic potential. Clin Cancer Res 2014; 20: 2136–2146. 24526730 10.1158/1078-0432.CCR-13-1939 PMC5902798

[bib53] Qiu Y, Zhou B, Su M, Baxter S, Zheng X, Zhao X et al. Mass spectrometry-based quantitative metabolomics revealed a distinct lipid profile in breast cancer patients. Int J Mol Sci 2013; 14: 8047–8061. 23584023 10.3390/ijms14048047 PMC3645730

[bib54] Paglia G, Hrafnsdottir S, Magnusdottir M, Fleming RM, Thorlacius S, Palsson BO et al. Monitoring metabolites consumption and secretion in cultured cells using ultra-performance liquid chromatography quadrupole-time of flight mass spectrometry (UPLC-Q-ToF-MS). Anal Bioanal Chem 2012; 402: 1183–1198. 22159369 10.1007/s00216-011-5556-4

