# Supplementary material for: Germline BAP1 mutations induce a Warburg effect
Source: Cell Death Differ. 2017 Jun 30;24(10):1694–704. doi: 10.1038/cdd.2017.95 (PMC5596430; doi:10.1038/cdd.2017.95)
Supplement: Supplementary Information [file cdd201795x1.docx]

**Supplemental Information**

**Germline *BAP1* mutations induce a Warburg effect**

Angela Bononi, Haining Yang, Carlotta Giorgi, Simone Patergnani, Laura Pellegrini, Mingming Su, Guoxiang Xie, Valentina Signorato, Sandra Pastorino, Paul Morris, Greg Sakamoto, Shafi Kuchay, Giovanni Gaudino, Harvey I. Pass, Andrea Napolitano, Paolo Pinton, Wei Jia, and Michele Carbone

**Supplemental Data**

Supplementary Figures

**Supplementary Figure 1**

OPLS-DA analysis of *BAP1^WT^* and *BAP1^+/-^* plasma metabolite profiles. An OPLS-DA model (N=61, K=412 variables including identified metabolites and unknowns) was constructed using plasma samples data (412 metabolites) from 37 *BAP1^WT^* subjects (blue dots) and 24 *BAP1^+/-^* (red dots). The OPLS-DA prediction model can separate *BAP1^WT^* and *BAP1^+/-^* individuals; plasma samples IDs of each sample are shown (see also Figure 1 and 2, and Supplementary Table 1). OPLS-DA, orthogonal partial least squares discriminant analysis.

**Supplementary Figure 2**

Hierarchical clustering and heat map showing 21 cell metabolites altered between *BAP1^WT^* and *BAP1^+/-^*, based on VIP ≥ 1.5. Colors from green to red represent the intensity of the metabolites from low (green) to high (red) levels in the analysis (see color scale). OPLS-DA, orthogonal partial least squares discriminant analysis; VIP, Variable Importance in the Projection.

**Supplementary Figure 3**

BAP1 silenced HM cells displayed increased glycolysis and reduced mitochondrial respiratory function. (**a**) WB of BAP1 protein levels in HM silenced for BAP1. HM were transfected with control scrambled siRNA or siBAP1 (a pool of 4 different siRNAs targeting BAP1: siBAP1#1, siBAP1#2, siBAP1#3 and siBAP1#5). (**b**) Extracellular acidification (ECAR) was measured using the Seahorse XF96 extracellular flux analyzer. ECAR provides a measurement of the glycolysis levels (scrambled: 22.24 ± 8.02 mpH/min; siBAP1 48.18 ± 9.09 mpH/min) and of the maximal glycolytic capacity (scrambled: 10.50 ± 3.20 mpH/min; siBAP1 26.93 ± 4.72 mpH/min). Data are means ± SEM of 3 independent experiments; * *P* < 0.05. (**c**) Oxygen consumption rate (OCR) was analyzed using the Seahorse XF96 extracellular flux analyzer. Bars depict the parameters measured to determine mitochondrial respiratory functions: basal respiration [OCR-BASAL (scrambled: 84.7 ± 9.34 pMoles/min; siBAP1 45.08 ± 10.51 pMoles/min)], ATP production [OCR-ATP (scrambled: 53.84 ± 7.35 pMoles/min; siBAP1 25.52 ± 6.71 pMoles/min)], maximal respiration [OCR-MRR (scrambled: 98.77 ± 7.70 pMoles/min; siBAP1: 47.27 ± 10.23 pMoles/min)], spare respiratory capacity [OCR-SRC (scrambled: 18.97 ± 6.93 pMoles/min; siBAP1: 5.83 ± 2.25 pMoles/min)]. Data are means ± SEM * *P* <0.05

**Supplementary Figure 4**

Mitochondrial morphology and mitochondrial membrane potential in *BAP1^WT^* and *BAP1*^+/-^ fibroblasts. (**a-d**) Mitochondrial morphology. *BAP1^WT^* and *BAP1*^+/-^ fibroblasts were transduced with an adenovirus expressing mitochondrial-targeted green fluorescent protein (mtGFP). Images acquired by digital microscopy were subjected to deconvolution, 3D reconstruction, and quantitatively analyzed. (**a**) Representative 3D reconstructed images of mitochondria in *BAP1^WT^* (left) and *BAP1*^+/-^ (right) fibroblasts transduced with mtGFP. (**b-d**) Bar graphs of (**b**) mitochondria number (*BAP1^WT^*: 126.57 ± 5.14 and *BAP1*^+/-^: 118.38 ± 4.84), (**c**) total mitochondrial volume (*BAP1^WT^*: 1961.26 ± 53.06 μm^3^ and *BAP1*^+/-^: 1993.92 ± 64.42 μm^3^), and (**d**) mean volume of single mitochondria volume (*BAP1^WT^*: 16.55 ± 0.81 μm^3^ and *BAP1*^+/-^: 17.62 ± 0.83 μm^3^). Data were obtained from 42 cells examined from three separate experiments. Data are expressed as mean ± SEM. (**e**) Mitochondrial membrane potential (ΔΨ). Cells were loaded with the potential-dependent probe tetramethyl rhodamine methyl ester (TMRM) and fluorescence was analyzed. Images on the left show TMRM-loaded mitochondria from *BAP1^WT^* and *BAP1*^+/-^ fibroblasts with Look-up Table rainbow-dark mask; colors from blue to red represent the intensity of the signal from low (blue) to high (red), and are directly proportional to ΔΨ. Bar graph on the right: depolarization rates were calculated as described in Supplemental Experimental Procedures.

Supplementary Tables

**Supplementary Table 1, related to Figure 1**.

Summary of genetic and demographic data, samples collected, and disease status, of the individuals involved in this study. LA: Louisiana family; WI: Wisconsin family; DOB: date of birth; DOD: date of death; MM: malignant mesothelioma; UM: uveal melanoma; MBAITs: Melanocytic BAP1-mutated Atypical Intradermal Tumors (benign melanocytic lesions, a specific phenotypic marker of *BAP1*-mutation carriers). We collected plasma samples from 46 individuals: 14 of the W family, 7 *BAP1^WT^* (3 males and 4 females) and 7 *BAP1^+/-^* (2 males and 5 females), and 32 of the L family, 23 were *BAP1^WT^* (9 males and 14 females) and 9 *BAP1^+/-^* (3 males and 6 females). A total of 61 plasma samples were collected over a period of 2 years, 37 from *BAP1^WT^* and 24 *BAP1^+/-^* individuals. We established 12 human dermal skin fibroblast cell cultures from skin punch biopsies, from 6 *BAP1^+/-^* carriers (3 from the W and 3 from the L families) and 6 *BAP1^WT^* control family members (3 from the W and 3 from the L families).

**Supplementary Table 2, related to Figure 1.**

Plasma metabolites. 71 plasma metabolites had VIP ≥ 1. Fold change (FC) was calculated as the ratio *BAP1^+/-^*/*BAP1^WT^*. FC with value larger than 1 indicates a higher level of the metabolite in *BAP1^+/-^* samples; a FC value lower than 1 indicates lower levels in *BAP1^+/-^* compared to *BAP1^WT^* samples. VIP: Variable Importance in the Projection.

**Supplementary Table 3, related to Figure 1.**

Cell metabolites. 111 cell metabolites had VIP ≥ 1. A FC (*BAP1^+/-^*/*BAP1^WT^*) value larger than 1 indicates a higher level of the metabolite in *BAP1^+/-^* samples. A FC value lower than 1 indicates a lower level in *BAP1^+/-^* compared to *BAP1^WT^* samples. FC, fold change; VIP, Variable Importance in the Projection.

**Supplementary Table 4, related to Figure 4.**

Mass distribution vectors (MDVs) and fractional contributions of ^13^C-glucose 6-P, ^13^C-citrate and ^13^C-lactate between *BAP1^WT^* and *BAP1^+/-^.*

**Supplementary Table 5**

Gene expression microarray.

RNA was extracted from *BAP1^WT^* and *BAP1^+/-^* fibroblast cell cultures and reversed transcribed to double-strand cDNA; cDNA products were then used as templates for *in vitro* transcription to generate fluorescent cRNA. Labeled cRNAs were hybridized to an Agilent SurePrint G3 Human GE v3 8x60K Microarray (Design ID: 072363). There are a total of 58,201 probes on Agilent SurePrint G3 Human GE v3 8x60K Microarray. Expression of genes coding for glycolysis, glycerol metabolism, pentose phosphate pathway, glycogen metabolism and TCA cycle enzymes was analyzed. Differentially expressed genes were determined using Welch’s *t*-test and Fold Change (FC) filtering. *P*-values were computed using asymptotic method and corrected using Benjamini-Hochberg FDR-multiple testing correction method.

**Supplemental Experimental Procedures**

**Subjects**

Germline *BAP1* sequencing was conducted on genomic DNA extracted from peripheral blood using standard methods and analyzed using bidirectional sequencing of the *BAP1* gene in our Hawaii Cancer Consortium CLIA/CAP certified laboratory. Primary cultures of human dermal fibroblasts were established from biopsies of sun-protected forearm skin obtained from subjects of different sexes and ages, after informed consent. Biopsies were taken at the Queen's Medical Center, using a 5 mm punch (Schuco) on skin site previously disinfected and anesthetized^1^.

**Cell cultures**

Fibroblasts were grown from skin explants and then cultured in Dulbecco modified Eagle's medium (DMEM) with glucose (4.5 g/l), 2mM L-glutamine, without sodium pyruvate (Corning, Cat. No. 10-017-CV), supplemented with 10% (v/v) FBS (Gibco) and 1% Penicillin-Streptomycin^1^. All the experiments were performed on fibroblasts between 7 and 15 passages of culture. Cells were cultured in a humidified atmosphere of 5% (v/v) carbon dioxide in air at 37 °C, and routinely tested for mycoplasma contamination. Primary human mesothelial cells (HM) were obtained (after informed consent) from pleural fluids of patients with non-malignant conditions, established in tissue culture, and characterized immunohistochemically, as routinely done in our laboratory^2^. HM cells were cultured in Dulbecco modified Eagle's medium (DMEM) with glucose (4.5 g/l), 2mM L-glutamine, without sodium pyruvate (Corning, Cat. No. 10-017-CV), supplemented with 20% (v/v) FBS (Gibco) and 1% Penicillin-Streptomycin.

**Plasma samples preparation and analysis by LC-TOF-MS**

A volume of 50 μL aliquot of plasma sample was spiked with 10 μL of aqueous 4-chlorophenylalanine (10 μg/mL, used as the internal standard) and 200 µL of a mixture of methanol and acetonitrile (5:3, v/v). The mixture was vortexed for 2 min, allowed to stand for 10 min, and then centrifuged at 13,000 rpm for 20 min. The supernatant was analyzed by LC-TOF-MS.

An Agilent HPLC 1200 system equipped with a binary solvent delivery manager and a sample manager (Agilent Corporation, Santa Clara, CA) was used with chromatographic separations performed on a 4.6 × 150 mm 5 μm Agilent ZORBAX Eclipse XDB-C18 chromatography column. The LC elution conditions were optimized as follows: isocratic at 1% B (0-0.5 min), linear gradient from 1% to 20% B (0.5-9.0 min), 20-75% B (9.0-15.0 min), 75-100% B (15.0-18.0 min), isocratic at 100% B (18–19.5 min); linear gradient from 100% to 1% B (19.5-20.0 min) and isocratic at 1% B (20.0–25.0 min) with a flow rate of 0.4 mL/min. The column was maintained at 30 °C. A 5 μL aliquot sample was injected into the column. Mass spectral data was acquired using an Agilent model 6220 MSD TOF mass spectrometer equipped with a dual sprayer electrospray ionization source (Agilent Corporation, Santa Clara, CA). The system was tuned for optimum sensitivity and resolution before analysis. Agilent API-TOF reference mass solution kit was used to obtain accurate mass time-of-flight data in both positive and negative mode operation. The TOF mass spectrometry was operated with the following optimized conditions: (1) ES+ mode, capillary voltage 3.5 kV, nebulizer 45 psig, drying gas temperature 325 °C, drying gas flow 11 L/min, and (2) ES- mode, similar conditions as ES+ mode except the capillary voltage was adjusted to 3.0 kV. During metabolite profiling, both plot and centroid data were acquired for each sample from 50 to 1,000 Da over a 25 min analysis time.

**Plasma samples preparation and analysis by UPLC-TQ-MS**

Each aliquot of plasma sample was extracted with 3-volume mixture of ethanol: chloroform (3:1 = v/v). The sample extracts were centrifuged at 4 °C and 14, 500 rpm for 20 min. The supernatant was used for targeted metabolic profiling of 140 lipids with an Acquity ultra performance liquid chromatography coupled to a Xevo TQ-S mass spectrometer (UPLC-TQ-MS, Waters Corp., Milford, MA). Briefly, each 10 µL of sample was directly injected into mass spectrometer with elution solvent (methanol with 5 mM ammonium acetate) at a varied flow rate from 30 to 200 µL/min within 3 min.

**Plasma samples preparation and analysis by GC-TOF-MS**

An aliquot of 50 μL plasma sample was spiked with two internal standard solutions (10 μl L-2-chlorophenylalanine in water, 0.3 mg/ml; 10 μL heptadecanoic acid in methanol, 1 mg/mL) and vortexed for 10 seconds. The mixed solution was extracted with 150 μL of methanol: chloroform (3:1) and vortexed for 30 seconds. After storing for 10 min at -20°C, the samples were centrifuged at 10,000 rpm for 10 min. An aliquot of the 150 μL supernatant was transferred to a glass sampling vial to vacuum dry at room temperature. The residue was derivatized using a two-step procedure. First, 80 μL methoxyamine (15 mg/mL in pyridine,) was added to the vial and kept at 30°C for 90 min, followed by 80 μL BSTFA (1%TMCS) at 70°C for 60 min.

Each 1 μl aliquot of derivatized solution was injected in splitless mode into an Agilent 7890N gas chromatograph coupled with a Pegasus HT TOF mass spectrometer (Leco Corporation). Separation was achieved on a DB-5 ms capillary column (30 m × 250 µm I.D., 0.25-µm film thickness; Agilent, J&W Scientific), with helium as the carrier gas at a constant flow rate of 1.0 ml/min. The temperature of injection, transfer interface, and ion source were set to 260°C, 260°C, and 210°C, respectively. The gas chromatography temperature programming was set to 2 min isothermal heating at 80°C, followed by 10°C/min oven temperature ramps to 220°C, 5°C/min to 240°C, and 25°C/min to 290°C, and a final 8 min maintenance at 290°C. Electron impact ionization (70 eV) at full scan mode (m/z 40-600) was used, with an acquisition rate of 20 spectra/sec in the TOF-MS setting.

**Cell extracts preparation and analysis by UPLC-TQMS and GC-TOF-MS**

The cell samples were prepared as previously described with modifications^3-5^. Appropriate weight of homogenizer beads and 50 µL of cold-water were added to the cell line samples for the first-step extraction. Each aliquot of 270-µL mixture of ethanol: chloroform (3:1 = v/v) was added to the extracts for second-step extraction. The sample extracts were centrifuged at 4 °C and 14, 500 rpm for 20 min.

**LC-TOF-MS Data analysis**

The acquired data files from LC-TOF-MS were processed using Agilent MassHunter Qualitative Analysis Program (vB.05.00, Agilent) and XCMS package, respectively. The acquired data files from UPLC-TQ-MS were processed with TargetLynx Application Manager (Waters Corp., Milford, MA) to extract peak area and retention time of each metabolite. The acquired data from GC-TOF-MS were analyzed by ChromaTOF software (v4.22, Leco Co.). Internal standards and any known artificial peaks, such as peaks caused by noise, column bleed and BSTFA derivatization procedure, were removed from the data set.

The four data sets resulting from GC-TOF-MS and LC-TOF-MS (ES+ and ES-) and UPLC-TQ-MS were combined and analyzed using multivariate statistical tools to establish characteristic metabolic profiles associated with different response phenotypes. Multivariate statistical analysis, orthogonal partial least squares-discriminant analysis (OPLS-DA), was performed with SIMCA-P 12.0 software (Umetrics, Umeå, Sweden). Variable importance in the projection (VIP) value was calculated to evaluate the contribution of individual metabolite on the OPLS-DA model^6^. Large VIP values greater than 1.0 are the most relevant for explaining differences between groups. In addition, the differential metabolites were further validated using a univariate statistical analysis, Student’s *t*-test.

For LC-MS, metabolite annotation was performed by comparing the accurate mass (*m/z*) and retention time (Rt) of reference standards in our in-house library and the accurate mass of compounds obtained from the web-based resources such as the Human Metabolome Database (<http://www.hmdb.ca/>).

For GC-MS, metabolite annotation was performed by comparing the mass fragments with NIST 05 Standard mass spectral databases in NIST MS search 2.0 (NIST) with a similarity of more than 70% and verified by available reference compounds.

**Gene expression analysis**

*RNA isolation* — Total RNA was extracted form *BAP1^WT^* and *BAP1^+/-^* using RNAprotect Cell Reagent (QIAGEN, Valencia, CA) according to the manufacturer’s instructions. Total RNA was further purified using the RNeasy Protect Cell Mini Kit (QIAGEN, Valencia, CA) according to the manufacturer’s protocol.

RNA quantity and quality were determined using a Nanodrop ND-1000 spectrophotometer (Thermo Fisher Scientific Inc., Waltham, MA) and an Agilent Bioanalyzer (Agilent Technologies, Santa Clara, CA).

*cRNA amplification and labeling* — Total RNA was amplified and labeled with Cyanine 3 (Cy3) using Agilent Low Input Quick Amp Labeling Kit, one-color (Agilent Technologies, Santa Clara, CA) following the manufacturer's instructions. Briefly, total RNA was reversed transcribed to double-strand cDNA using a poly dT-T7 promoter primer. Primer, template RNA and quality-control transcripts of known concentration and quality were first denatured at 65 ℃ for 10 min and incubated for 2 hours at 40°C with 5X first strand Buffer, 0.1 M DTT, 10 mM dNTP mix, and AffinityScript RNase Block Mix. The AffinityScript enzyme was inactivated at 70°C for 15 min. cDNA products were then used as templates for in vitro transcription to generate fluorescent cRNA. cDNA products were mixed with a transcription master mix in the presence of T7 RNA polymerase and Cy3 labeled-CTP and incubated at 40°C for 2 hours. Labeled cRNAs were purified using QIAGEN’s RNeasy mini spin columns and eluted in 30 μl of nuclease-free water. After amplification and labeling, cRNA quantity and cyanine incorporation were determined using a Nanodrop ND-1000 spectrophotometer and an Agilent Bioanalyzer.

*Sample hybridization* — For each hybridization, 0.60 μg of Cy3 labeled cRNA were fragmented, and hybridized at 65 ℃ for 17 hours to an Agilent SurePrint G3 Human GE v3 8x60K Microarray (Design ID: 072363). After washing, microarrays were scanned using an Agilent DNA microarray scanner.

*Data analysis of microarray* — Intensity values of each scanned feature were quantified using Agilent feature extraction software version 11.5.1.1, which performs background subtractions. We only used features that were flagged as no errors (detected flags), and excluded features that were not positive, not significant, not uniform, not above background, saturated, and population outliers (not detected and compromised flags). Normalization was performed using Agilent GeneSpring software version 13.1.1 (per chip:normalization to 75 percentile shift). There are a total of 58,201 probes on Agilent SurePrint G3 Human GE v3 8x60K Microarray (Design ID: 072363) without control probes.

Agilent GeneSpring software was used for statistical analysis of the data after microarrays scanning. We compared relative difference of gene expression of measured genes between the groups of 6 *BAP1^+/-^* as compared to 6 *BAP1^WT^* individuals. Differentially expressed genes were determined using Welch’s t test and Fold Change (FC) filtering. P-values were computed using asymptotic method and corrected using Benjamini-Hochberg FDR-multiple testing correction method.

**Western blot**

Total cell lysates were prepared in M-PER (Thermo Scientific, cat. no. 78501) reagent supplemented with proteases and phosphatases inhibitors (2 mM Na_3_VO_4_, 2 mM NaF, 50 nM Okadaic Acid (OA), 1 mM PMSF and protease inhibitor cocktail), and 1 mM DTT. When indicated, cells were silenced for 24 hours or transduced with adenoviruses for 36 hours. Proteins extracts were quantified using the Bradford assay (Bio-Rad Laboratories), 7 μg of proteins were loaded and separated on NuPAGE® Novex 4-12% Bis-Tris Gel (Life Technologies), and electron-transferred to PVDF membrane according to standard procedures. Antibodies used were: α-Tubulin (4G1) (Santa Cruz Biotechnology, cat. no. sc-58666), BAP1 (D7W7O) (Cell Signaling, cat. no. 13271).

**XF96 instrument setup and analysis**

*BAP1^WT^* and *BAP1^+/-^* fibroblasts were seeded (6 replicates) in XF96 Cell Culture Microplates (Seahorse Bioscience) at 3×10^4^ cells/well in 200 µL of DMEM 10% FBS. HM were seeded at 40x10^3^ cells well in 200 μl DMEM 10% FBS.

Cells were incubated for 24 hours at 37 °C in 5% CO_2_ atmosphere, and then transfected with siRNAs-BAP1 or scrambled using HiPerFect Transfection Reagent (Qiagen). Alternatively, fibroblasts were transduced with adenoviruses for wild type BAP1 protein (AdBAP1) or its catalytic inactive mutant carrying the C91S point mutation (AdC91S).

Before the experiments, the culture medium was removed from each well and replaced by 175 μl of unbuffered Seahorse XF Base Medium base (cat. no. 102353-100 Agilent Technologies) pre-warmed at 37 °C, supplemented with 10 mM glucose, 1 mM Pyruvate and 2 mM glutamine (for analysis of mitochondrial oxidative metabolism) or 2 mM glutamine (for analysis of glycolysis assessment), pH 7.4. Cells were incubated in a CO_2_ free incubator at 37 °C for 1 h. Prior to the rate measurements, the XF96 Analyzer (Seahorse biosciences, North Billerica, MA) automatically mixed the assay media in each well for 10 min to allow the oxygen partial pressure to reach equilibrium. Each measurement cycle consisted of a mixing time of 2 minutes and a data acquisition period of 4 minutes (12 data points). ECAR (extracellular acidification rate) and OCR (oxygen consumption rate) data points refer to the average rates during the measurement cycles and were reported as absolute rates (mpH/min for ECAR, pmoles/min for OCR). The following compounds were prepared at appropriate concentrations as reported below, and adjusted to pH 7.4. ECAR measurements: 5 mM Glucose, 2 mM Oligomycin A, 50 mM 2-DG; OCR measurements: 2 μm Oligomycin, 1 μm FCCP 1 uM, 1 μm Rot/AntA. A volume of 25 μL of compound was added to each injection port, and 3 baseline measurements were taken prior to the addition of any compound. Then, after a 3 min wait, 3 response measurements were taken after each addition.

ECAR and OCR values were normalized to the number of cells per well using the crystal violet method; briefly, immediately after measurements, cells were fixed with 2% paraformaldehyde for 10 min. After 3 washes in PBS, cells were stained with 0.1 % crystal violet for 20 min and washed. Finally, absorbance was measured at 595 nm.

**ATP assay**

Fibroblasts were seeded at 5 × 10^3^ cells/96-well in white tissue culture-treated sterile microplate. After 72 hours, total levels of cellular ATP were determined using a Luminescent ATP Detection Assay Kit (Abcam, Cat. No. ab113849) according to the manufacturer’s protocol.

**Lactate assay**

Fibroblasts were grown to full confluence in cell-culture treated 96-well plates. After 24 hours, the amount of released lactate was determined using a colorimetric L-Lactate Assay Kit (Abcam) according to the manufacturer’s protocol.

**Mitochondrial morphology analysis**

Cells were seeded onto 24-mm glass coverslips, allowed to grow to 50–60% confluence and then transduced with an adenovirus expressing mitochondrial-targeted green fluorescent protein (mtGFP). After 36 hours, cells were imaged with a Nikon Swept Field confocal microscope equipped with a CFI Plan Apo VC60XH objective (n.a. 1.4) (Nikon Instruments) and an Andor DU885 EM-CCD camera (Andor Technology Ltd). The coverslip were placed in an incubation chamber with controlled temperature, CO_2_ and humidity, and then 51-plane z-stacks were acquired with a voxel dimension of 133x133x200 nm (X x Y x Z). Mitochondrial parameters analyzed as number and volume were determined using the 3D object counter available in software Fiji (http://fiji.sc/wiki/index.php/Fiji). Mitochondria 3D renders were obtained with the 3D Viewer plugin.

**Mitochondrial membrane potential**

Mitochondrial membrane potential was measured by loading cells with 20 nM tetramethyl rhodamine methyl ester (TMRM) for 30 min at 37°C. Images were obtained on an inverted microscope (Nikon LiveScan Swept Field Confocal Microscope Eclipse Ti). TMRM excitation was performed at 560 nm and emission was collected through a 590 to 650 nm band-pass filter. Images were acquired every 5 s with a fixed 20 ms exposure time. FCCP (carbonyl cyanide p-trifluoromethoxyphenylhydrazone, 10 μM), an uncoupler of oxidative phosphorylation, was added after 12 acquisitions to completely collapse the electrical gradient established by the respiratory chain. Data are shown as depolarization rate between the final fluorescence intensity (after FCCP addition) and the initial fluorescence intensity (before FCCP addition).

**Statistical analysis**

Statistical analyses were performed using Student’s *t*-tests, unless otherwise specified. *P*-values < 0.05 were considered statistically significant and marked with asterisks (**P* < 0.05; ***P* < 0.01), as indicated in the Figure legends. Data are represented as mean ± SEM.

**Supplementary References**

1. Bononi, A. *et al.* BAP1 regulates IP3R3-mediated Ca2+ flux to mitochondria suppressing cell transformation. *Nature* (in press).

2. Yang, H. *et al.* TNF-alpha inhibits asbestos-induced cytotoxicity via a NF-kappaB-dependent pathway, a possible mechanism for asbestos-induced oncogenesis. *Proc Natl Acad Sci U S A* **103**, 10397-402 (2006).

3. Qiu, Y. *et al.* A distinct metabolic signature of human colorectal cancer with prognostic potential. *Clin Cancer Res* **20**, 2136-46 (2014).

4. Qiu, Y. *et al.* Mass spectrometry-based quantitative metabolomics revealed a distinct lipid profile in breast cancer patients. *Int J Mol Sci* **14**, 8047-61 (2013).

5. Minni, A. *et al.* Long-term (12 to 18 months) functional voice assessment to detect voice alterations after thyroidectomy. *Eur Rev Med Pharmacol Sci* **18**, 1704-8 (2014).

6. Galindo-Prieto, B., Eriksson, L. & Trygg, L. Variable influence on projection (VIP) for orthogonal projections to latent structures (OPLS). *Journal of Chemometrics* **28**, 623–632 (2014).
