# Supplementary figures and images for: Germline BAP1 mutations induce a Warburg effect
Source: Cell Death Differ. 2017 Jun 30;24(10):1694–704. doi: 10.1038/cdd.2017.95 (PMC5596430; doi:10.1038/cdd.2017.95)

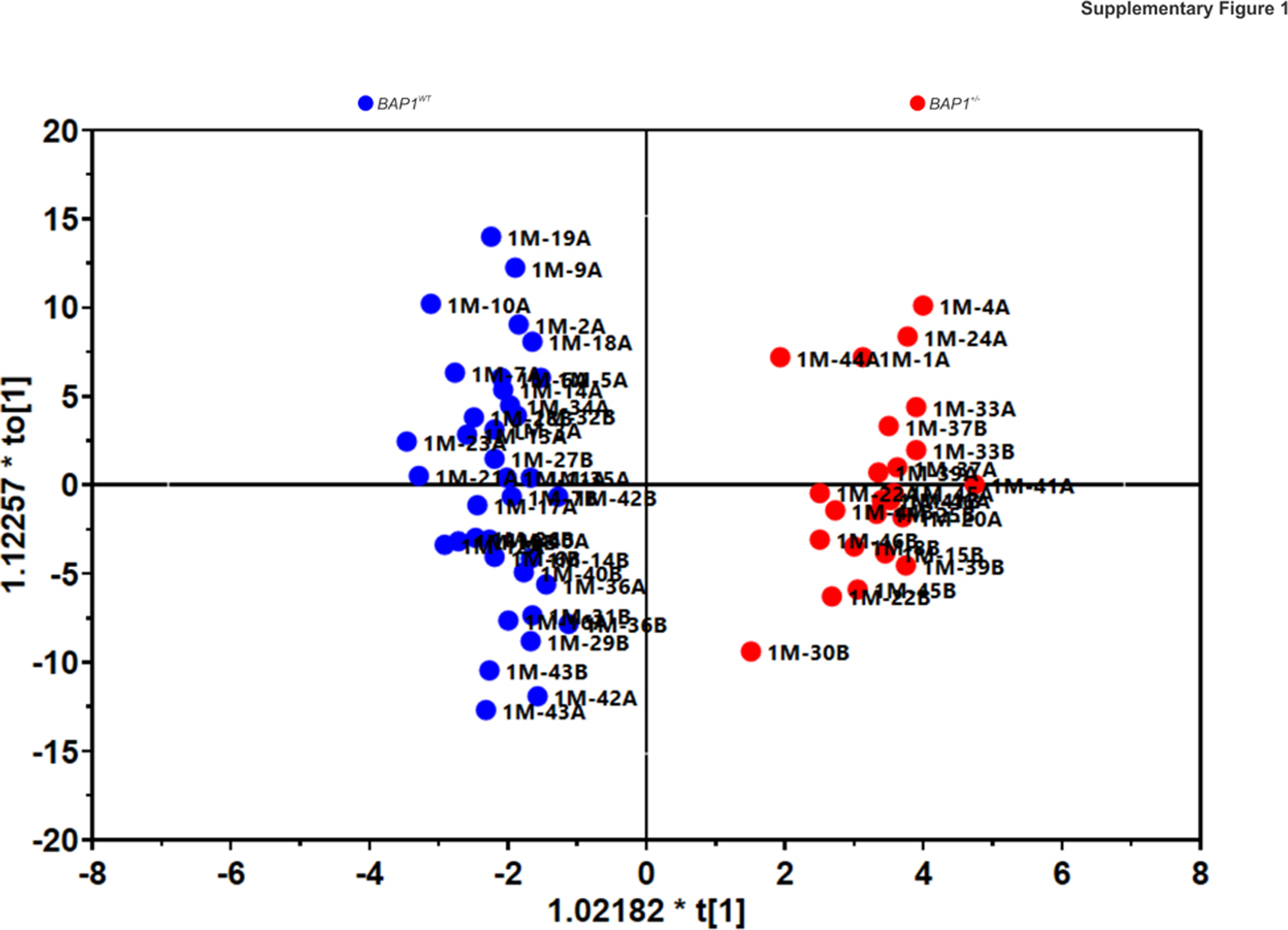

Supplement: Supplementary Figure 1 [file cdd201795x2.tif]

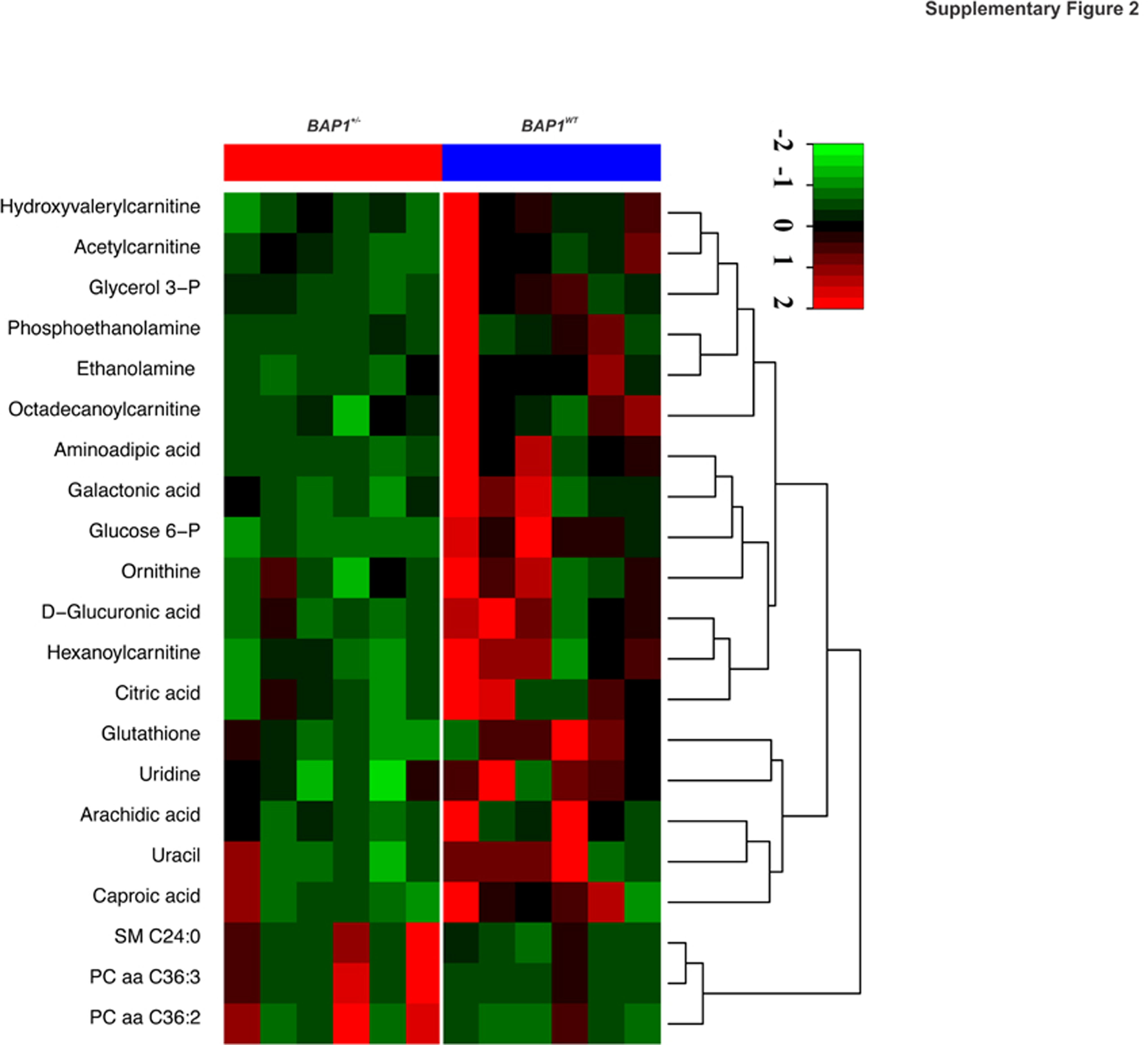

Supplement: Supplementary Figure 2 [file cdd201795x3.tif]

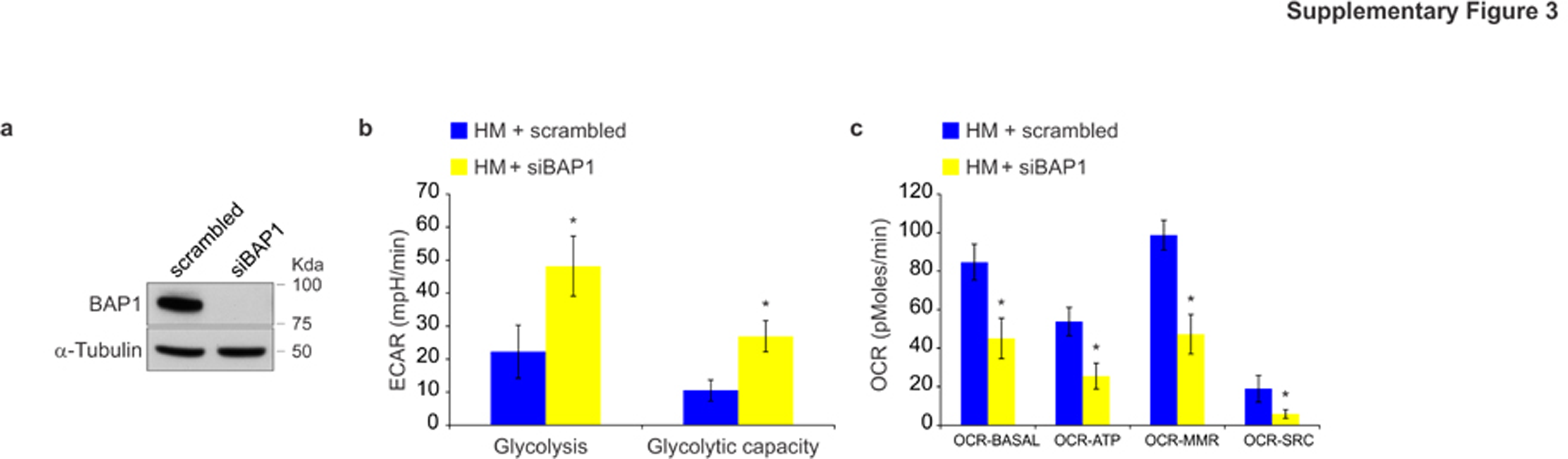

Supplement: Supplementary Figure 3 [file cdd201795x4.tif]

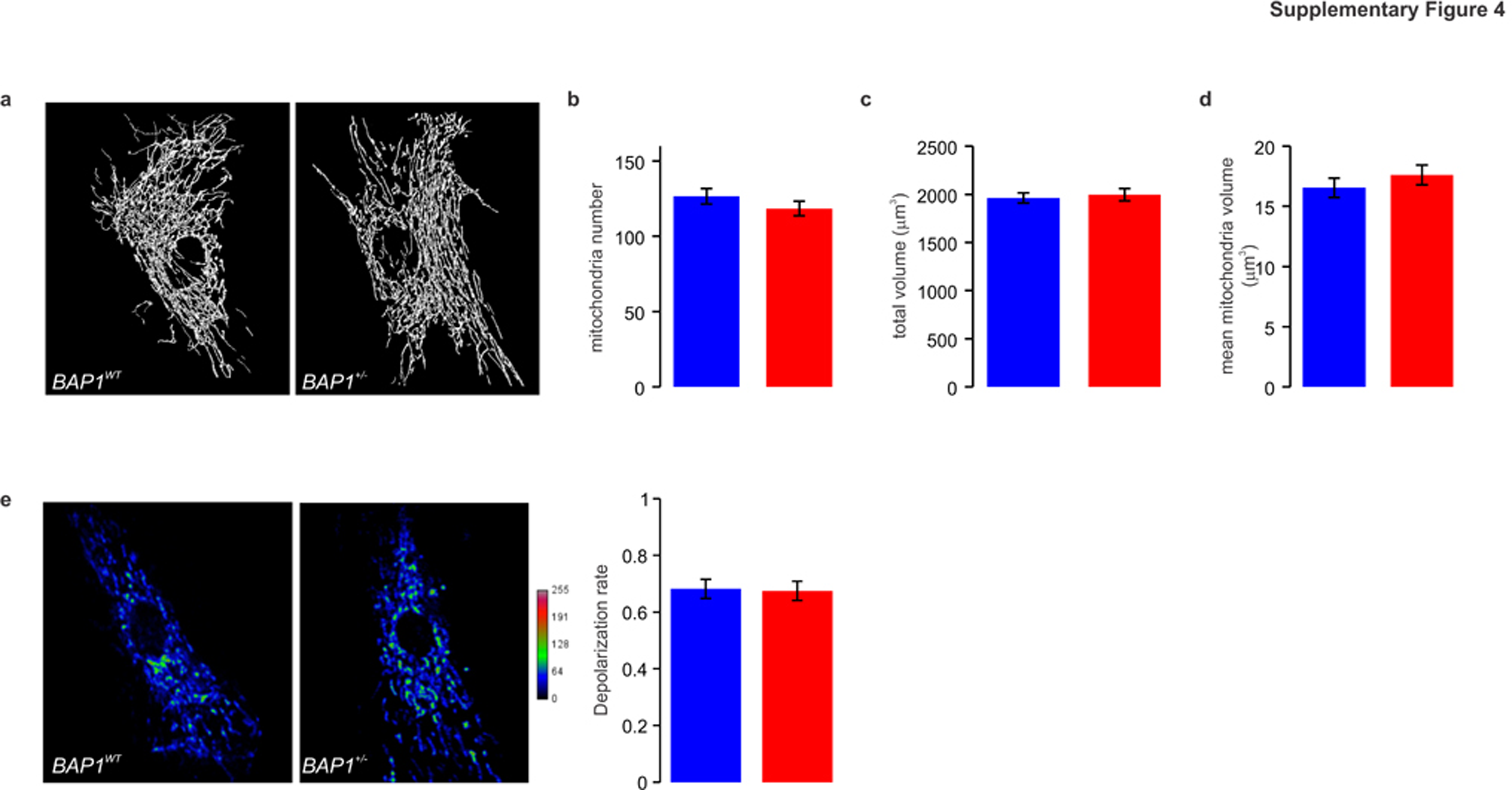

Supplement: Supplementary Figure 4 [file cdd201795x5.tif]
