# Supplementary material for: Germline BAP1 mutations induce a Warburg effect
Source: Cell Death Differ. 2017 Jun 30;24(10):1694–704. doi: 10.1038/cdd.2017.95 (PMC5596430; doi:10.1038/cdd.2017.95)
Supplement: Supplementary Table 4 [file cdd201795x9.docx]

**Supplementary Table 4. Mass distribution vectors (MDVs) and fractional contributions of ^13^C-glucose 6-P, ^13^C-citrate and ^13^C-lactate between *BAP1^WT^* and *BAP1^+/-^***

|  | | **^13^C glucose 6-P** | | **^13^C-citrate** | | **^13^C-lactate** | |
| --- | --- | --- | --- | --- | --- | --- | --- |
|  |  | ***BAP1^WT^*** | ***BAP1^+/-^*** | ***BAP1^WT^*** | ***BAP1^+/-^*** | ***BAP1^WT^*** | ***BAP1^+/-^*** |
| **MDV** | **M+0** | 0.7301 | 0.8682 | 0.1706 | 0.1715 | 0.2654 | 0.2838 |
|  | **M+1** | 0.0000 | 0.0000 | 0.1069 | 0.1087 | 0.2316 | 0.2529 |
|  | **M+2** | 0.0000 | 0.0000 | 0.6884 | 0.6882 | 0.2575 | 0.2597 |
|  | **M+3** | 0.0000 | 0.0000 | 0.0083 | 0.0099 | 0.2455 | 0.2643 |
|  | **M+4** | 0.0000 | 0.0000 | 0.0151 | 0.0160 |  |  |
|  | **M+5** | 0.0000 | 0.0000 | 0.0061 | 0.0017 |  |  |
|  | **M+6** | 0.2699 | 0.1318 | 0.0046 | 0.0041 |  |  |
| **Fractional contributions** | | 0.2699 | 0.1318 | 0.2712 | 0.2686 | 0.3708 | 0.3913 |
